# Development and validation of an interpretable machine learning model for predicting medium-to-giant coronary aneurysms in Kawasaki disease

**DOI:** 10.3389/fimmu.2026.1804276

**Published:** 2026-03-24

**Authors:** Jiaying Zhang, Jing Li, Jinfeng Dong, Tingjiao You, Miao Hou, Ying Liu, Ying He, Panpan Liu, Yinghao Wang, Lizhi Li, Shurong Ma, Haitao Lv, Hongbiao Huang

**Affiliations:** 1 Department of Cardiology, Children’s Hospital of Soochow University, Suzhou, Jiangsu, China; 2 Department of Hematology, The First Affiliated Hospital of Fujian Medical University, Fuzhou, Fujian, China; 3 Department of Hematology, National Regional Medical Center, The First Affiliated Hospital of Fujian Medical University, Fuzhou, Fujian, China; 4 Institute of Pediatric Research, Children’s Hospital of Soochow University, Suzhou, Jiangsu, China; 5 Department of Pediatrics, Fujian Provincial Hospital, Fuzhou University Affiliated Provincial Hospital, Fuzhou, Fujian, China; 6 Department of Pediatric Surgery, Fujian Provincial Hospital, Fujian Provincial Clinical Medical College of Fujian Medical University, Fuzhou, Fujian, China; 7 Department of Endocrinology, Children’s Hospital of Soochow University, Suzhou, Jiangsu, China

**Keywords:** artificial intelligence, coronary artery aneurysm, kawasaki disease, machine learning, risk assessment

## Abstract

**Purpose:**

This study aimed to develop and validate an interpretable machine learning (ML) model using routinely collected clinical data to predict medium-to-giant coronary artery aneurysms (MGCAA) early in Kawasaki disease (KD).

**Methods:**

This retrospective study included 2,777 KD patients from two centers in China. Eleven ML algorithms were developed using clinical and laboratory data from electronic medical records (EMRs). Recursive feature elimination and SHapley Additive exPlanations (SHAP) were used for feature selection and interpretability. The final model was internally and externally validated, with intercept-only recalibration to correct miscalibration, and evaluated by area under the receiver operating characteristic curve (AUC), calibration, and decision curve analysis (DCA). The model was deployed as an R Shiny–based online prediction tool.

**Results:**

The support vector machine model implemented with kernlab (SVM kernlab) included seven key features: time to diagnosis, monocyte percentage, rash, eosinophil percentage, C-reactive protein, triglycerides, and neutrophil percentage. It achieved an AUC of 0.732 (95% CI, 0.597–0.866) in internal validation and 0.689 (95% CI, 0.611–0.767) in external validation. SHAP analysis provided both global feature importance and individualized explanations. Recalibration improved model calibration, and DCA demonstrated meaningful net clinical benefit across clinically relevant threshold probabilities.

**Conclusion:**

This study presents an interpretable ML model to predict MGCAA risk in KD using routine clinical data, supporting clinical risk assessment.

## 1 Introduction

Kawasaki disease (KD) is an acute, self-limited vasculitis that primarily affects small- and medium-sized arteries, with a particular predilection for the coronary arteries (1). It mainly affects children under 5 years and shows marked geographic, ethnic, and seasonal variation, with the highest incidence in East Asian populations (2). Timely administration of intravenous immunoglobulin (IVIG) has significantly reduced the incidence of coronary artery aneurysms (CAA), from approximately 25% to 3–5% (3). While mild dilatations and small CAAs often resolve spontaneously (4), medium-to-giant coronary artery aneurysms (MGCAA) are associated with a markedly higher risk of long-term cardiovascular complications, including thrombosis, myocardial infarction, and sudden death (1, 5). These patients often require prolonged follow-up and aggressive treatment, including dual antiplatelet or anticoagulant therapy (1, 6). Early identification of patients at high risk for MGCAA is therefore critical for timely intervention and improved outcomes.

Several prognostic models for KD have been developed to predict overall coronary artery involvement or IVIG resistance (7–10). Only a limited number of studies have attempted to identify MGCAA-specific risk factors (11, 12).However, the lack of adequate external validation in these studies limits their generalizability. In addition, the absence of formal sample size calculations in most prediction model studies raises concerns about potential overfitting and restricted transportability (13). Moreover, the use of echocardiography as the primary diagnostic tool poses challenges in resource-limited settings, as it depends heavily on operator expertise and availability (14).

In recent years, machine learning (ML) approaches have shown promise in clinical risk modeling, particularly for their ability to handle high-dimensional data and uncover nonlinear relationships (15–17). However, the “black-box” nature of many ML models has limited their adoption in clinical practice, as the decision-making processes are often opaque to clinicians (18). To address this issue, explainable artificial intelligence techniques such as SHapley Additive exPlanations (SHAP) have been increasingly used to enhance model transparency, providing both global and patient-specific interpretations (19, 20).

This study developed and validated an interpretable ML model to predict MGCAA in children with KD using routine clinical data from two centers, incorporated model recalibration, and provided SHAP-based interpretability with a web tool for individualized risk assessment.

## 2 Materials and methods

The study was conducted in accordance with the principles of the Declaration of Helsinki. This study was approved by the Ethics Committee of the Children’s Hospital of Soochow University (Approval No. 2024CS165) and the Ethics Committee of Fujian Provincial Hospital (Approval No. K2025-02-105). This retrospective study used fully de-identified data extracted from electronic medical records (EMRs); all personal identifiers were removed prior to analysis to ensure patient confidentiality. Written informed consent was obtained from the participants’ legal guardians. The study was reported in accordance with the Transparent Reporting of a Multivariable Prediction Model for Individual Prognosis or Diagnosis plus Artificial Intelligence (TRIPOD+AI) statement (**Supplementary Table 1**) (21).

### 2.1 Study design and population

This retrospective study included pediatric KD patients from two tertiary hospitals in China, with a development cohort from Children’s Hospital of Soochow University (March 1, 2019–June 30, 2024) and an external validation cohort from Fujian Provincial Hospital (May 1, 2012–May 31, 2024), diagnosed according to American Heart Association (AHA) criteria (1, 6) and meeting predefined inclusion and exclusion criteria. MGCAA were defined according to the AHA guideline criteria (1, 6), with coronary artery Z-scores calculated using the Dallaire (22) method based on pre-IVIG echocardiography, and were considered the positive outcome of this study.

### 2.2 Data collection and preprocessing

Clinical and laboratory predictors measured prior to IVIG were extracted from EMRs. The Suzhou cohort was randomly split into training (70%) and internal validation (30%) sets using a fixed seed. Missing data were handled using multiple imputation by chained equations (MICE) (23), and multicollinearity was assessed.

### 2.3 Machine learning model development

Eleven ML algorithms were trained using repeated cross-validation within the training set. Class imbalance was addressed using Random Over-Sampling Examples (ROSE) (24). applied only to the training data, and model selection was based on the highest mean area under the receiver operating characteristic curve (AUC) across resampling folds.

### 2.4 Feature selection and model interpretation

Feature importance was assessed using SHAP, which provided both global importance rankings and individual-level interpretability. To refine the model, recursive feature elimination (RFE) was applied to identify the optimal subset of predictors (25).

### 2.5 Recalibration and clinical utility assessment

To address miscalibration caused by ROSE-altered event prevalence, an intercept-only recalibration was applied using internal validation data, and model performance before and after recalibration was assessed by observed-to-expected (O/E) ratio, calibration slope, Brier score. Decision curve analysis (DCA) assessed clinical utility across threshold probabilities.

### 2.6 Web-based tool deployment

The final model was deployed as an R Shiny–based online tool that provides real-time individualized MGCAA risk estimates, with the optimal decision threshold determined by the Youden index to support clinical risk assessment.

### 2.7 External validation

Minimum sample size was estimated using pmvalsampsize (26).An intercept-only recalibration was then performed in the external dataset, and performance before and after recalibration was evaluated using AUC, O/E ratio, calibration slope, and Brier score.

### 2.8 Quality, risk of bias, and applicability assessment

Methodological quality, risk of bias, and applicability were evaluated using the Prediction model Risk Of Bias Assessment Tool for Artificial Intelligence (PROBAST+AI) across four domains in both the model development and evaluation phases, with domain-level and overall judgments determined according to predefined signalling-question rules (27).

### 2.9 Statistical analysis

All statistical analyses were conducted using R version 4.3.1 and SPSS version 25.0 (IBM, Armonk, New York, USA). Normality of continuous variables was assessed using the Shapiro–Wilk test. Normally distributed data are presented as mean ± standard deviation and compared using t-tests, while non-normally distributed variables are reported as median (interquartile range) and analyzed using the two-tailed Mann–Whitney U test. Categorical variables were compared using chi-square or Fisher’s exact tests, as appropriate. The DeLong test was used to compare AUCs between models. *P*-value < 0.05 was considered statistically significant.

Detailed methods are provided in the **Supplementary Materials**.

## 3 Results

### 3.1 Correlation analysis

**Supplementary Table 2** lists variables that were available at only one center and were therefore excluded. **Supplementary Figure 1** presents the Spearman correlation heatmap of the retained variables. Most variables show weak correlations, indicating minimal multicollinearity. Red denotes positive correlations and blue denotes negative correlations, with darker colors representing stronger relationships. Hierarchical clustering is included to help identify groups of related variables.

Due to high collinearity (|r| > 0.60), repetitive urine test parameters—including bilirubin (BIL), urobilinogen (URO), ketone bodies (KET), occult blood (BLD), protein (PRO), nitrite (NIT), specific gravity (SG), urine pH (UpH), urine crystallization, and urine clarity index (UCI)—were removed. More clinically interpretable and reproducible urinary microscopic parameters, namely urine white blood cell count (UWBC) and urine red blood cell count (URBC), were retained. Similarly, derived hematological indices—including mean corpuscular hemoglobin concentration (MCHC), red cell distribution width (RDW), mean platelet volume (MPV), and platelet distribution width (PDW)—were excluded due to redundancy with their corresponding primary measures, hemoglobin (Hb) and platelet count (PLT). The proportion of missing data for the remaining variables is presented in **Supplementary Table 3**.

### 3.2 Baseline characteristics

**Figure 1:**
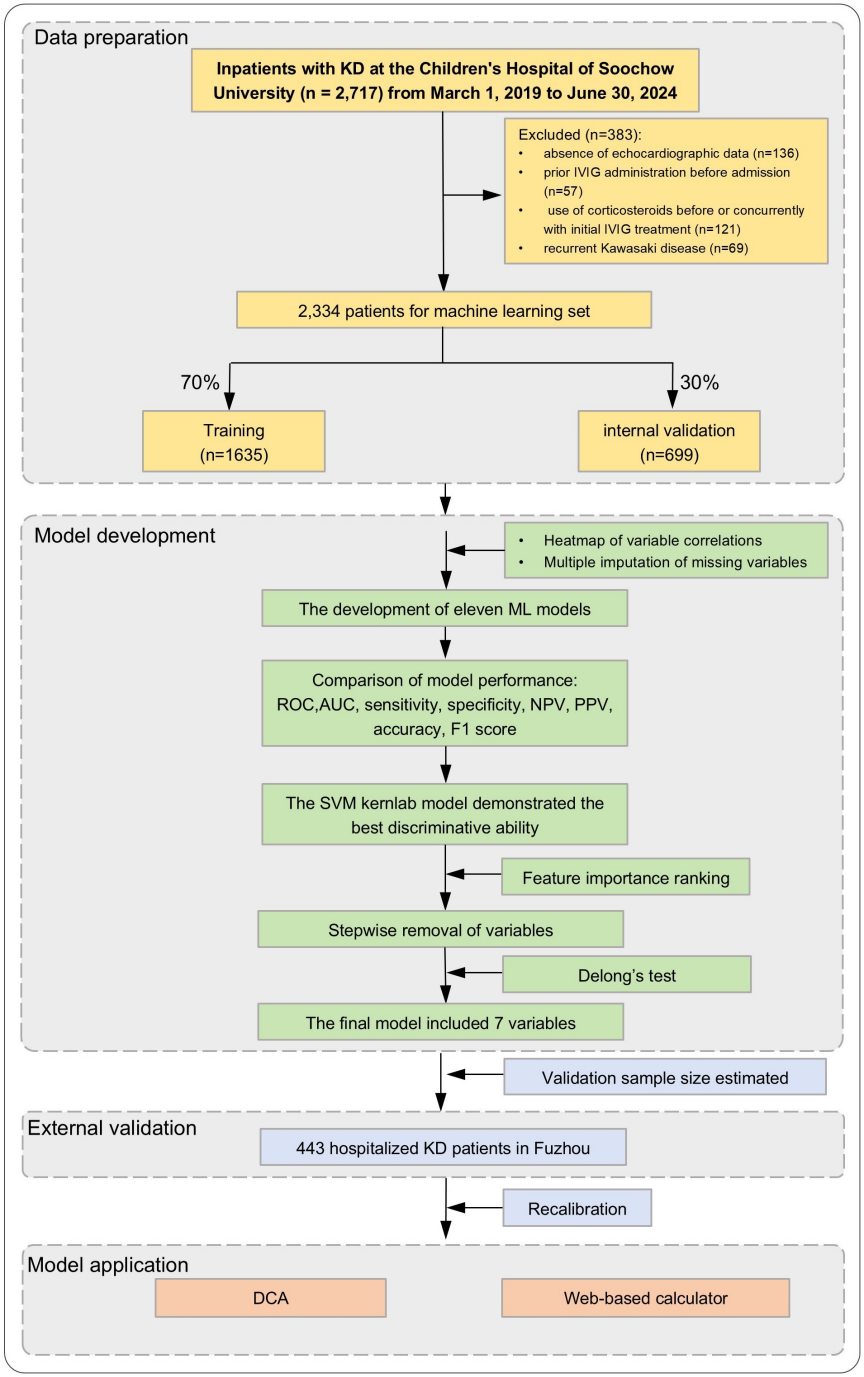
Flowchart of the study design and analysis. KD, Kawasaki disease; IVIG, intravenous immunoglobulin; ML, machine learning; ROC, receiver operating characteristic; AUC, area under the curve; NPV, negative predictive value; PPV, positive predictive value; SVM Kernlab, support vector with kernlab algorithm; DCA, decision curve analysis.

The patient selection process is depicted in **Figure 1**. Within the Suzhou cohort (n = 2,334), patients were classified into two groups based on the extent of coronary artery involvement: the non-MGCAA group (n = 2,279) and the MGCAA group (n = 55). **Table 1** presents the comparative analysis of demographic characteristics, clinical features, and laboratory parameters between these two groups. Significant differences were observed in variables such as time to diagnosis, conjunctival injection, oral mucosal changes, rash, cervical lymphadenopathy, white blood cell (WBC), PLT, Hb, serum albumin (ALB), creatine kinase (CK), serum sodium (Na), and UWBC.

**Table 1:** Comparison of clinical characteristics between patients with and without MGCAA in the Suzhou cohort.

Characteristics	Non-MGCAA (n = 2279)	MGCAA (n = 55)	*P* value
Male, n (%)	1377 (60.4)	39 (70.9)	.116
Age at presentation, months	26.00 (14.00, 47.00)	21.00 (10.00, 47.00)	.150
Time to diagnosis, days	6.00 (5.00, 7.00)	8.00 (6.00, 10.00)	<.001*
Conjunctival injection, n (%)	2029 (89.0)	40 (72.7)	<.001*
Oral mucosal change, n (%)	1989 (87.3)	40 (72.7)	.002*
Edema of the hands and feet, n (%)	1047 (45.9)	21 (38.2)	.254
Rash, n (%)	1714 (75.2)	32 (58.2)	.004*
Cervical lymphadenopathy, n (%)	2032 (89.2)	41 (74.5)	.001*
Perianal desquamation, n (%)	197 (8.6)	5 (9.1)	>.999
WBC, ×10^9^/L	12.31 (8.83, 16.23)	15.91 (10.45, 19.50)	.002*
NEU%	60.70 (44.10, 74.70)	51.90 (38.30, 68.50)	.089
EOS%	2.00 (0.60, 4.00)	2.30 (0.80, 4.40)	.233
MONO%	6.40 (4.70, 8.30)	7.60 (4.80, 9.50)	.109
PLT, ×10^9^/L	363.00 (284.00, 459.00)	446.00 (339.00, 579.00)	<.001*
Hb, g/L	113.00 (107.00, 120.00)	109.00 (102.00, 116.00)	<.001*
LYMPH%	29.30 (17.70, 43.80)	34.50 (22.00, 46.70)	.163
CRP, mg/L	64.07 (37.41, 100.97)	64.00 (40.03, 119.32)	.544
TG, mmol/L	1.16 (0.93, 1.48)	1.22 (1.00, 1.49)	.287
TC, mmol/L	3.65 (3.15, 4.15)	3.81 (3.19, 4.43)	.279
LDH, U/L	354.50 (297.50, 426.10)	374.50 (287.10, 473.80)	.616
GLU, mmol/L	5.40 (4.80, 6.20)	5.30 (4.70, 5.70)	.098
ALB, g/L	40.00 (37.60, 42.00)	39.30 (36.00, 40.90)	.022*
Ca, mmol/L	2.19 (1.10, 2.34)	2.14 (1.10, 2.31)	.659
CK, U/L	60.70 (41.60, 88.90)	45.60 (29.30, 63.20)	<.001*
AST, U/L	33.90 (26.30, 50.20)	32.10 (25.90, 48.50)	.581
ALT, U/L	22.40 (13.60, 67.10)	20.80 (13.30, 50.40)	.652
Cr, μmol/L	24.70 (20.40, 30.30)	23.80 (18.10, 30.20)	.287
GGT, U/L	20.00 (11.70, 72.00)	30.50 (15.00, 69.80)	.067
Mg, mmol/L	0.99 (0.93, 1.05)	1.01 (0.93, 1.06)	.511
Na, mmol/L	135.00 (133.00, 137.00)	136.00 (134.00, 138.00)	.030*
K, mmol/L	3.90 (3.60, 4.20)	4.00 (3.70, 4.30)	.325
Cl, mmol/L	103.00 (102.00, 105.00)	103.00 (101.00, 105.00)	.415
ALP, U/L	175.00 (147.00, 222.00)	174.00 (143.00, 221.00)	.808
BUN, mmol/L	3.04 (2.40, 3.75)	3.09 (2.40, 4.32)	.465
CKMB, ng/ml	1.20 (0.79, 1.90)	1.20 (0.60, 2.10)	.642
UWBC,/μL	7.40 (2.40, 24.00)	3.50 (1.00, 8.40)	.003*

Continuous variables are presented as median (interquartile range); categorical variables as numbers and percentages. * *P* <.05.

MGCAA, medium-to-giant coronary artery aneurysms; WBC, white blood cell; NEU%, neutrophil percentage; EOS%, eosinophil percentage; MONO%, monocyte percentage; PLT, platelet count; Hb, hemoglobin; LYMPH%, lymphocyte percentage; CRP, C-reactive protein; TG, triglyceride; TC, total cholesterol; LDH, lactate dehydrogenase; GLU, glucose; ALB, serum albumin; CK, creatine kinase; AST, aspartate aminotransferase; ALT, alanine aminotransferase; Cr, creatinine; GGT, γ-glutamyl transpeptidase; ALP, alkaline phosphatase; BUN, blood urea nitrogen; CKMB, creatine kinase-MB; UWBC, urinary white blood cell.

In the Fuzhou cohort, 400 patients were categorized as non-MGCAA and 43 as MGCAA. The comparative analysis of clinical and laboratory characteristics between these two groups is provided in **Supplementary Table 4**. Inter-center differences in baseline characteristics between the Suzhou and Fuzhou cohorts are summarized in **Supplementary Table 5**.

A stratified analysis of the entire cohort (N = 2,777) was also conducted based on the severity of coronary artery involvement. Patients were categorized into five groups: no involvement (n = 1,915), mild dilatation (n = 317), small CAA (n = 447), medium CAA (n = 88), and large or giant CAA (n = 10). The detailed stratified characteristics across these subgroups are presented in **Supplementary Table 6**.

### 3.3 Model development

**Figure 2:**
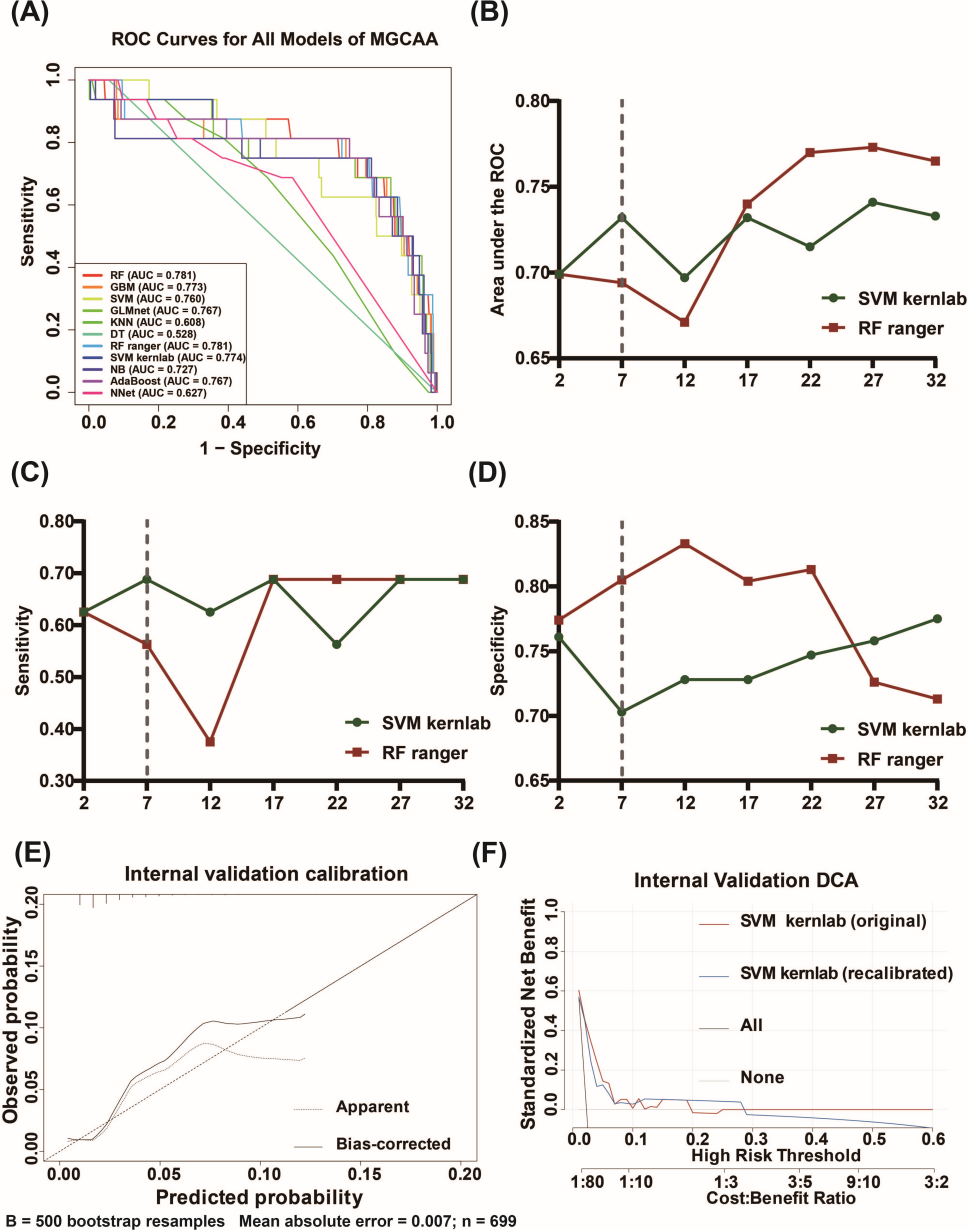
Model performance comparison and evaluation of feature reduction, calibration, and clinical utility for MGCAA prediction. **(A)** ROC curves of the 11 ML models; **(B)** AUC values with stepwise reduction in the number of features; **(C)** Sensitivity with stepwise feature reduction; **(D)** Specificity with stepwise feature reduction. **(E)** Internal validation calibration curve of the final SVM kernlab model with bootstrap bias correction. **(F)** DCA in the internal validation cohort comparing the original and recalibrated SVM kernlab models with “treat all” and “treat none” strategies. MGCAA, medium-to-giant coronary artery aneurysms; AUC, area under the receiver operating characteristic curve; DCA, decision curve analysis; GBM, gradient boosting machine; SVM kernlab, support vector machine with kernlab algorithm; KNN, K-nearest neighbor; RF ranger, random forest with ranger algorithm; GLMnet, generalized linear model via elastic net; Adaboost, adaptive boosting; NB, naive Bayes; DT, decision tree; RF, random forest; SVM, support vector machine; NNET, neural network.

**Supplementary Table 7** shows that ROSE increased MGCAA in the training set from 39/1635 (2.39%) to 802/1635 (49.05%), while the test and external validation sets remained unaltered. A total of eleven ML models were developed to predict the risk of MGCAA in KD patients using the training set. Key performance metrics, including AUC, sensitivity, specificity, positive predictive value (PPV), negative predictive value (NPV), accuracy, and F1 score, are summarized in **Table 2** and visualized in **Figure 2A**. The top-performing models were Random Forest (RF) (AUC = 0.781), RF with ranger implementation (RF ranger) (AUC = 0.781), and SVM with kernlab package (SVM kernlab) (AUC = 0.774).

**Table 2:** Predictive performance of 11 ML models for MGCAA in KD.

Model	AUC	Sensitivity	Specificity	PPV	NPV	Accuracy	F1 score
RF	0.781	0.839	0.688	0.991	0.091	0.835	0.909
GBM	0.773	0.628	0.813	0.993	0.049	0.632	0.770
SVM	0.760	0.946	0.313	0.983	0.119	0.931	0.964
GLMnet	0.767	0.792	0.688	0.991	0.072	0.790	0.880
KNN	0.608	0.170	0.938	0.991	0.026	0.187	0.290
DT	0.528	0.057	0.999	0.999	0.024	0.079	0.108
RF ranger	0.781	0.770	0.750	0.992	0.071	0.770	0.867
SVM kernlab	0.774	0.799	0.750	0.993	0.081	0.798	0.886
NB	0.727	0.818	0.688	0.991	0.081	0.815	0.897
Adaboost	0.767	0.641	0.813	0.993	0.050	0.645	0.779
NNET	0.627	0.102	0.938	0.986	0.024	0.122	0.186

ML, machine learning; MGCAA, medium-to-giant coronary artery aneurysms; KD, Kawasaki Disease; AUC, area under the receiver operating characteristic curve; PPV, positive predictive value; NPV, negative predictive value; RF, random forest; GBM, gradient boosting machine; SVM, support vector machine; GLMnet, generalized linear model with elastic net regularization; KNN, K-nearest neighbor; DT, decision tree; RF ranger, random forest with ranger algorithm; SVM kernlab, support vector machine with kernlab algorithm; NB, naive Bayes; Adaboost, adaptive boosting; NNET, neural network.

Among these, the SVM kernlab and RF ranger models were selected for further refinement, as both met the predefined thresholds of AUC, sensitivity, specificity, accuracy, and F1 score, all exceeding 0.700. Feature importance was assessed using SHAP values (**Supplementary Figure 2**), followed by stepwise elimination of less informative variables. The impact of feature reduction on model performance (AUC, sensitivity, specificity) was detailed in **Supplementary Table 8** and illustrated in **Figures 2B–D**. With feature counts ranging from 12 to 2, the SVM kernlab model outperformed the RF ranger model, with AUC improvements ranging from 0.026 to 0.038; therefore, the SVM kernlab model was selected for subsequent analysis.

The optimal model was a linear SVM implemented using the *svmLinear* method from the *kernlab* library. Hyperparameter tuning was performed using a predefined grid of cost parameters (C = (0.1, 1, 10)), and the final model selected C = 1 based on internal cross-validation. This model used a linear kernel, included 1,172 support vectors, achieved a training error of 0.305, and enabled probability estimation to generate individualized predicted risks.

A DeLong test confirmed that the 7-feature SVM kernlab model (AUC = 0.732, 95% CI, 0.597–0.866) exhibited no significant performance decline compared to the full 35-feature model (AUC = 0.746, 95% CI, 0.589–0.904) (Z = −0.484, *P* = 0.629). Moreover, the 7-feature model achieved a significantly higher AUC than the 12-feature model (AUC = 0.697, 95% CI, 0.545–0.849) (Z = 2.192, *P* = 0.028). While the difference with the 2-feature model (AUC = 0.699, 95% CI, 0.515–0.883) was not statistically significant (Z = 0.458, *P* = 0.647), the 7-feature model demonstrated a higher AUC (ΔAUC = 0.033) and superior overall performance.

Considering its optimal balance between predictive accuracy, model simplicity, and clinical applicability, the 7-feature SVM kernlab model was selected as the final model. The seven variables included time to diagnosis, monocyte percentage (MONO%), rash, eosinophil percentage (EOS%), C-reactive protein (CRP), triglycerides (TG), and neutrophil percentage (NEU%). The final model achieved an AUC of 0.732(95% CI, 0.597–0.866), with a sensitivity of 0.688 and specificity of 0.703 in the internal validation cohort.

### 3.4 Model calibration and decision curve analysis

Because oversampling altered the baseline event prevalence, recalibration was required to correct absolute risk estimates. Before recalibration, the O/E was 0.054 (95% CI, 0.033–0.090), the Brier score was 0.201, and the calibration slope was close to 0.94 (95% CI, 0.43–1.46). After intercept-only recalibration, the O/E ratio improved to 1.00 (95% CI, 0.50–2.00), the Brier score markedly decreased to 0.022, and the calibration slope remained unchanged (0.94, 95% CI, 0.43–1.46). The calibration plot (**Figure 2E**) showed good agreement between predicted and observed probabilities within the 0–0.10 risk range, particularly across the clinically relevant decision region where most patients were located. However, at higher predicted probabilities, the bias-corrected curve fell below the ideal diagonal line, indicating systematic overestimation of risk in the high-risk range.

In this study, when the threshold probability ranged from 0.01 to 0.28, the model provided greater net clinical benefit than either the “treat all” or “treat none” strategies. When the threshold exceeded 0.28, the model offered no additional net benefit compared with the “treat none” strategy (**Figure 2F**).

### 3.5 Model explanation

**Figure 3:**
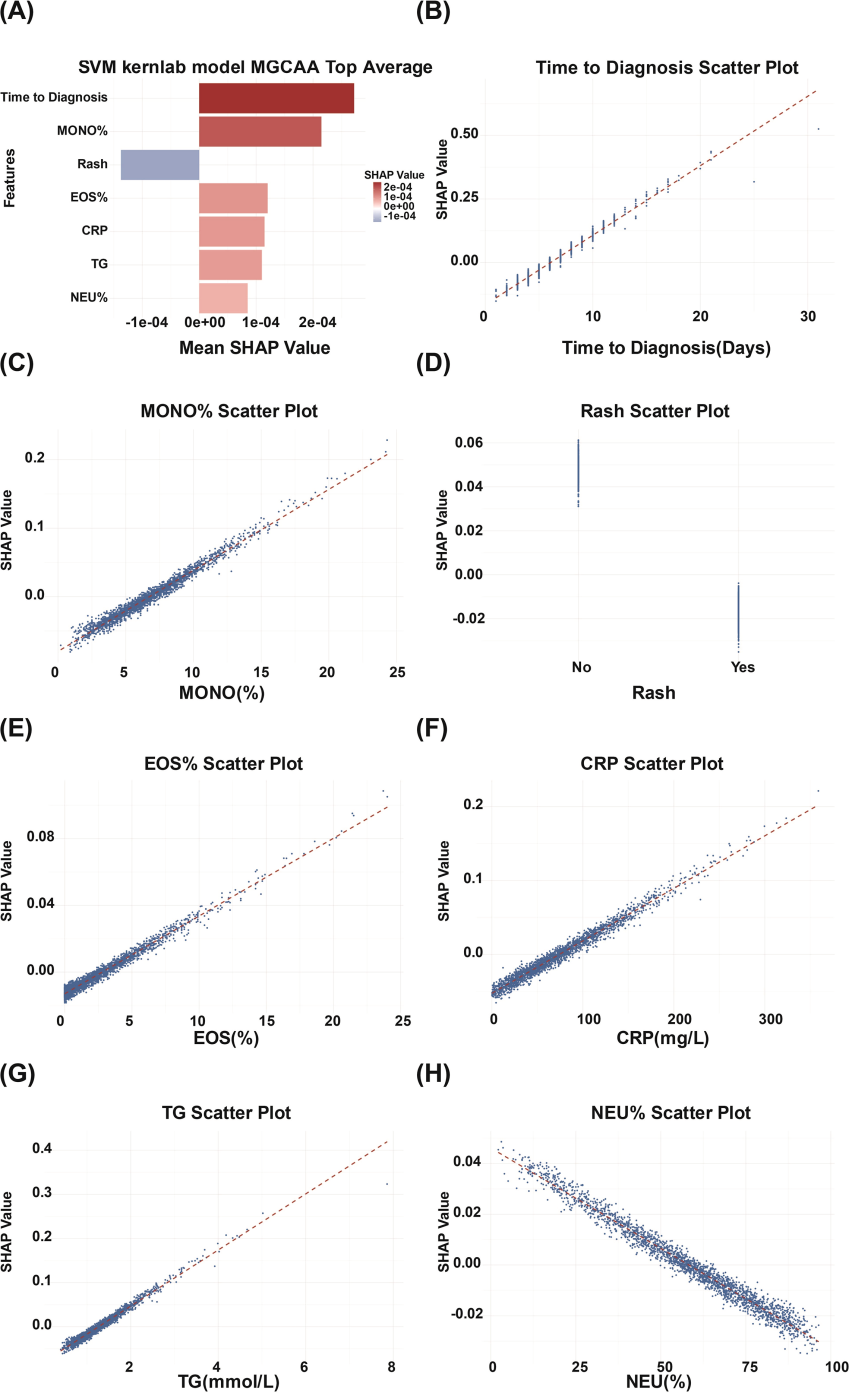
SHAP-based global feature importance and individual variable effects in the SVM kernlab model. **(A)** Top seven features ranked by SHAP values in the SVM kernlab model; **(B–H)** SHAP scatter plots of individual key features. SHAP, SHapley Additive exPlanation; MGCAA, medium-to-giant coronary artery aneurysms; MONO%, monocyte percentage; EOS%, eosinophil percentage; CRP, C-reactive protein; TG, triglycerides; NEU%, neutrophil percentage.

Global feature importance was assessed using the SHAP method, as illustrated in **Figure 3A**. Predictive variables were ranked according to their average SHAP values, representing their relative contributions to the model’s output. Among these, time to diagnosis and MONO% emerged as the most influential predictors in determining MGCAA risk. **Figures 3B–H** depict the distribution of actual variable values alongside their corresponding SHAP values for each of the seven selected features. A SHAP value greater than zero indicates an increased likelihood of developing MGCAA, while values below zero suggest a reduced risk, reflecting a potentially protective association. For instance, when MONO% exceeds 7.9% or CRP levels surpass 96.03 mg/L, the SHAP values are positive, signifying elevated risk. Conversely, the presence of a rash consistently corresponds to negative SHAP values, indicating a protective effect against MGCAA development.

**Figure 4:**
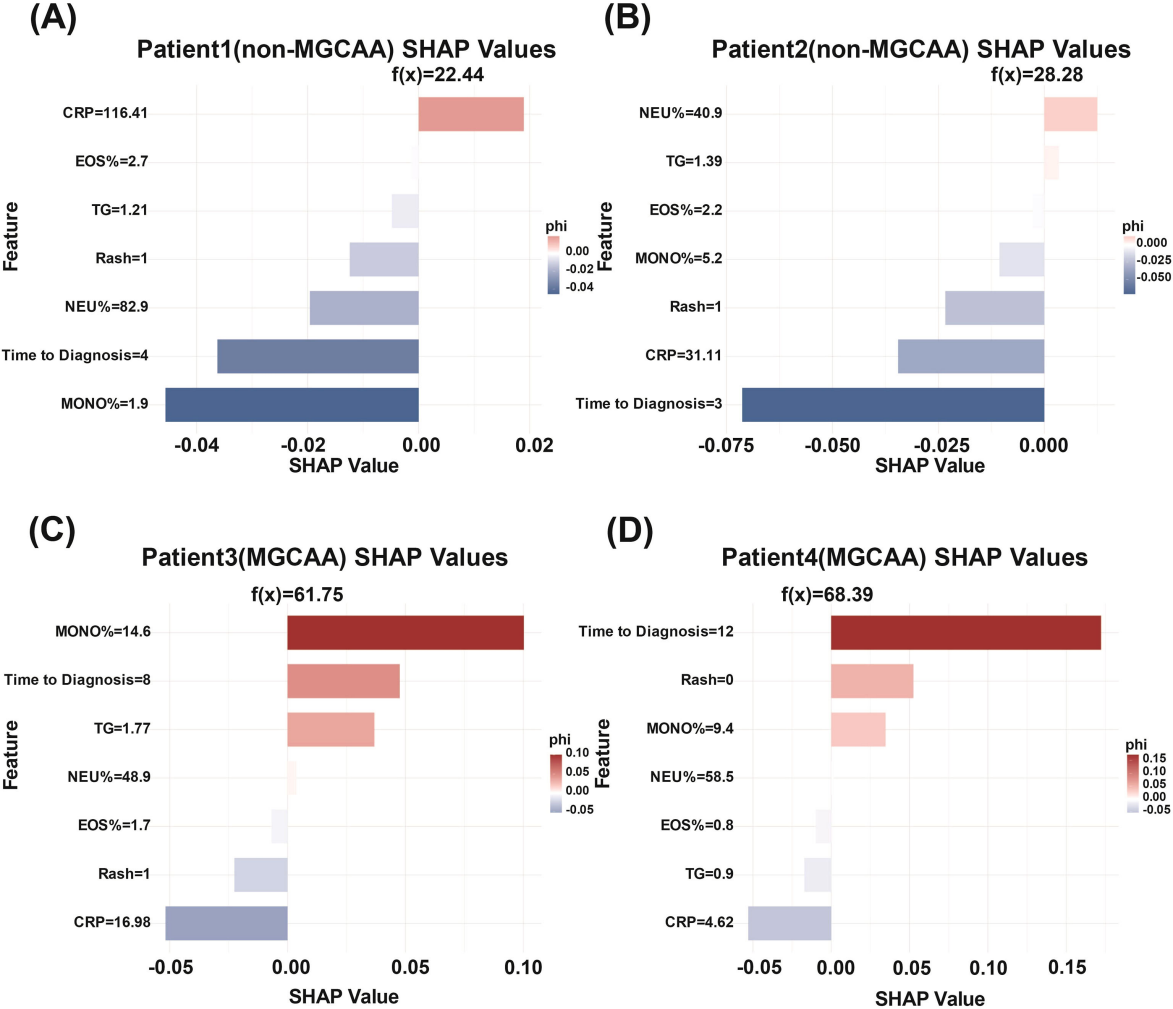
SHAP-based local explanations for four individual patients. SHAP, SHapley Additive exPlanation; MGCAA, medium-to-giant coronary artery aneurysms; MONO%, monocyte percentage; EOS%, eosinophil percentage; CRP, C-reactive protein; TG, triglycerides; NEU%, neutrophil percentage.

In addition to global interpretability, SHAP was applied for individualized explanation of model predictions. **Figure 4** presents the local interpretability analysis for four representative patients, two diagnosed with MGCAA and two without.

### 3.6 Clinical application

**Figure 5:**
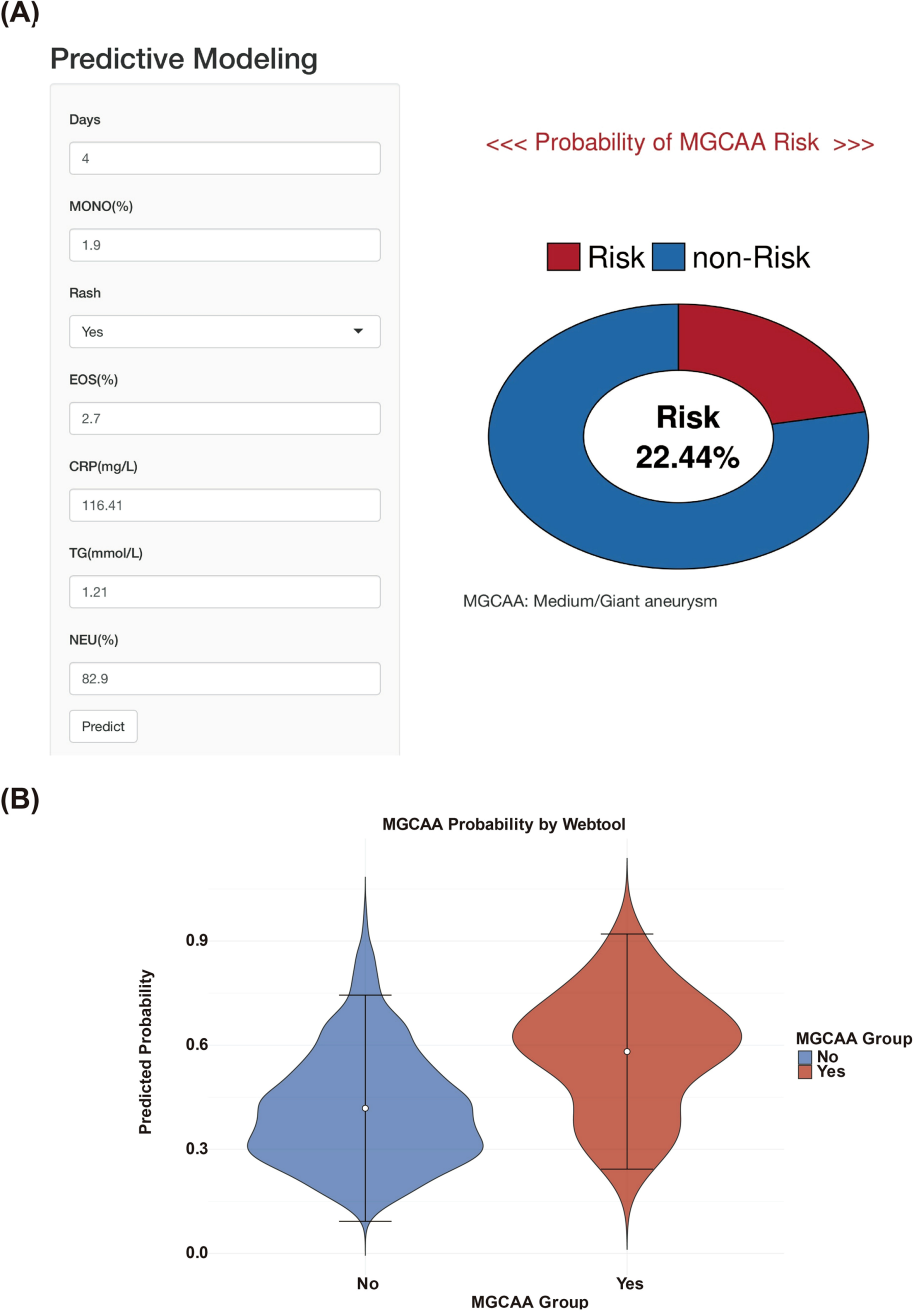
Online MGCAA risk calculator interface and probability distribution visualization. **(A)** Web-based prediction tool developed using R Shiny; **(B)** Predicted risk probabilities for MGCAA and non-MGCAA groups. MGCAA: medium-to-giant coronary artery aneurysms; MONO%: monocyte percentage; EOS%: eosinophil percentage; CRP: C-reactive protein; TG: triglycerides; NEU%: neutrophil percentage.

The finalized SVM kernlab prediction model was deployed as an interactive, web-based platform to facilitate clinical application (**Figure 5A**). This user-friendly tool allows clinicians to input real-time values for seven key clinical indicators of pediatric patients, after which the system computes and displays the individualized probability of developing MGCAA. If any required predictor is missing, the calculation will be halted to ensure the reliability of the prediction. The optimal cut-off derived from the model yielded a Youden index of 50.2%, providing a practical reference point to assist clinicians in risk stratification. The tool is publicly accessible at: https://medium-giant-aneurysm.shinyapps.io/cals/. **Figure 5B** illustrates the distribution of predicted risk probabilities across the non-MGCAA and MGCAA groups, highlighting the model’s discriminative capability.

### 3.7 External validation

Based on the observed outcome prevalence in the external cohort (9.7%), we calculated the minimum sample size for external validation to be 330 participants with at least 33 events using the pmvalsampsize package, under the assumption of a normally distributed linear predictor (mean = -0.17, SD = 0.73). **Supplementary Figure 3** illustrates the simulated distribution of the linear predictor used in this calculation. Our actual external validation cohort included 443 individuals and 43 events, meeting the sample size requirements.

**Figure 6:**
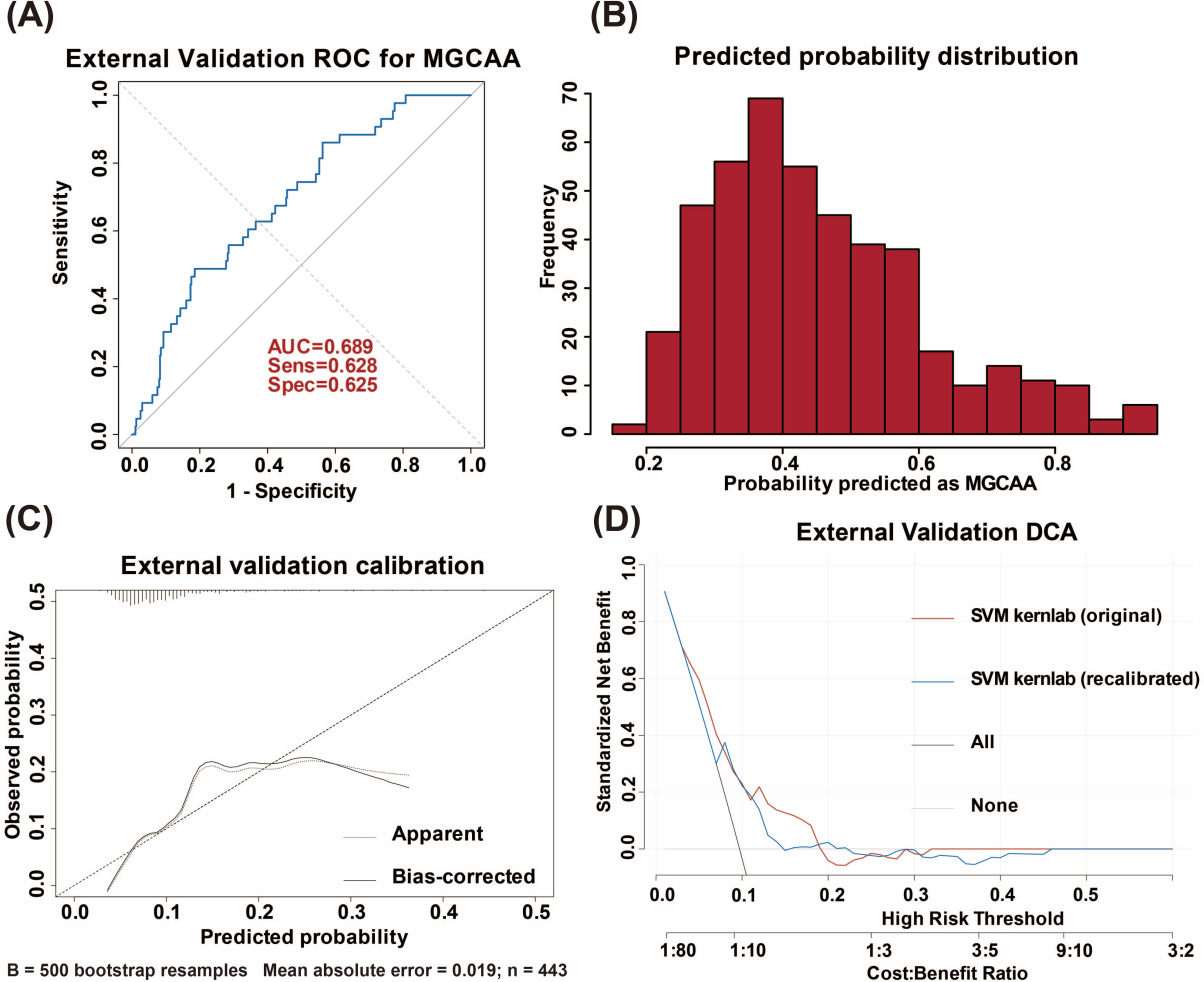
External validation of the model. **(A)** Model performance evaluation in the external validation cohort. **(B)** The predicted probability distribution in the external validation cohort. **(C)** Calibration curve with bootstrap bias correction in the external validation cohort. **(D)** DCA comparing the original and recalibrated SVM kernlab models with “treat all” and “treat none” strategies. ROC, receiver operating characteristic; AUC, area under the curve; MGCAA, medium-to-giant coronary artery aneurysms; DCA, decision curve analysis; SVM kernlab, support vector machine with kernlab algorithm.

In the independent Fuzhou cohort, the SVM kernlab model showed moderate discrimination (AUC = 0.689; 95% CI, 0.611–0.767; sensitivity = 0.628; specificity = 0.625(**Figure 6A**). **Figure 6B** illustrates the distribution of uncalibrated predicted probabilities for MGCAA in this cohort. The distribution is shifted toward higher predicted probabilities, indicating systematic overestimation of absolute risk before recalibration.

Before recalibration, the O/E was 0.21 (95% CI, 0.15–0.30), the Brier score was 0.241, and the calibration slope was 0.72 (95% CI, 0.35–1.09). After intercept-only recalibration, the O/E ratio improved to 1.00 (95% CI, 0.66–1.53) and the Brier score decreased to 0.089. The calibration slope showed minimal change (0.69, 95% CI, 0.29–0.99). Discrimination, as measured by the AUC, remained unchanged. When predicted probabilities exceed 0.20, the bias-corrected curve falls below the diagonal, indicating overestimation of MGCAA risk in the moderate-to-high range (**Figure 6C**).

In the external validation cohort, threshold probabilities between 0.07 and 0.22 corresponded to a higher net clinical benefit for the model compared with both the “treat all” and “treat none” strategies. Above a threshold of 0.22, the model offers no added benefit over “treat none” (**Figure 6D**).

### 3.8 PROBAST +AI assessment

The PROBAST+AI assessment indicated high risk of bias in the participants domain due to the retrospective design, and in the predictors domain because blinding of predictor assessment to outcome status could not be ensured. The outcomes and analysis domains were judged as low risk, and applicability across participants, predictors, and outcomes showed low concern (**Supplementary Table 9**).

## 4 Discussion

### 4.1 Summary of key findings

In this multicenter study, MGCAA incidence was 2.4% in the Suzhou cohort and 9.7% in the Fuzhou cohort, indicating substantial inter-center heterogeneity. Previous studies suggest that regional variation in KD-related coronary complications may be associated with differences in population genetic background and variability in the timeliness and accuracy of KD recognition and clinical management across centers (12, 28, 29). However, the underlying causes of the heterogeneity observed in our cohorts remain unclear and warrant further investigation.

Multiple ML algorithms were constructed and compared, with the SVM kernlab algorithm demonstrating the most favorable performance. The SVM kernlab model employed in this study utilizes kernel functions to project input features into high-dimensional space, enabling the identification of an optimal hyperplane that maximizes classification margins. This approach makes the model particularly suitable for capturing nonlinear relationships in high-dimensional clinical datasets (14, 30). The final 7-feature SVM kernlab model achieved an AUC of 0.732 (95% CI, 0.597–0.866) in internal validation and 0.689 (95% CI, 0.611–0.767) in external validation. After recalibration, the AUC of our model remained unchanged, supporting its potential value for clinical risk assessment of MGCAA among KD patients.

### 4.2 Sample size adequacy for model validation

Small sample sizes increase the risk of overfitting and bias, especially in flexible machine learning models that typically require larger datasets (31, 32). In accordance with recent methodological recommendations for clinical prediction model validation, we performed a rigorous sample size estimation based on the distribution of the linear predictor (logit of predicted probabilities) (26, 33). Our external validation cohort (N = 443, with 43 events) exceeded the calculated minimum requirement (N = 330, 33 events). This supports the credibility and generalizability of our model performance results in the external dataset. However, it should be noted that while the overall sample size was adequate, the number of events remained relatively low, which may compromise the stability of certain performance metrics.

### 4.3 Comparison with previous studies

Long-term outcomes vary significantly based on aneurysm size, Persistent giant CAAs remain the most serious complications of KD. Genetic studies have identified several loci associated with coronary artery outcomes in KD, including inositol 1,4,5-trisphosphate 3-kinase C (ITPKC), Fc gamma receptor IIA (FCGR2A), caspase 3 (CASP3), CD40, and interleukin-1 beta (IL-1β), supporting a potential role of host genetic susceptibility (34). Some studies have further suggested that the progression of coronary artery complications in KD may be independent of infectious factors (35, 36).

A 15-year follow-up study demonstrated that small CAAs generally resolved without leading to stenotic lesions, whereas medium-sized CAAs had a stenosis incidence of 58% (5). Similarly, a Japanese follow-up study focusing on giant CAAs reported event-free survival rates of 80% at 1 year, 72% at 5 years, and 68% at 10 years (37). Medium-sized CAAs require prolonged follow-up and dual antiplatelet therapy, while giant CAAs are associated with higher risks of myocardial infarction, thrombosis, and aneurysm rupture, necessitating combined anticoagulant therapy (1). MGCAA demand more intensive clinical management, highlighting the urgent need for specialized prediction tools.

ML has been applied to predict IVIG resistance and coronary artery lesions in KD; however, small sample sizes, class imbalance, and the lack of multicenter validation continue to limit its clinical applicability (38). To date, only two studies have developed MGCAA-specific prediction models. Jiang et al. identified six independent predictors (male sex, older age, prolonged fever, IVIG resistance, elevated PLT, and hypoalbuminemia) via logistic regression analysis (12). Zhao et al. reported that prolonged fever, IVIG resistance, cardiomegaly, elevated pre-treatment white blood cell (≥ 12.05×10^9^/L), hypoalbuminemia (≤ 37.25 g/L), and reduced neutrophil change (ΔN ≤ 30.2%) were associated with MGCAA development (11). These models fail to capture complex nonlinear relationships among predictors and lack sufficient external validation, underscoring the need for a more robust MGCAA prediction model.

### 4.4 Predictor variables

Through SHAP-based feature importance ranking and comparative analysis of AUC values across models with varying numbers of features, a final predictive model incorporating seven variables was selected: time to diagnosis, MONO%, rash, EOS%, CRP, TG, and NEU%. Among these, prolonged time to diagnosis emerged as the most significant predictor of MGCAA. Delayed diagnosis is a well-established risk factor for CAA, often associated with older age, atypical clinical presentation, incomplete KD, and earlier admission periods (39, 40). Notably, van Stijn et al. reported that each one-day delay in IVIG treatment increases the odds of medium and giant CAA by 10% and 20%, respectively (41). Such delays are believed to sustain inflammatory responses, leading to progressive vascular wall injury and subsequent aneurysm formation.

Elevated monocyte levels, as key mediators of inflammation, may contribute to vascular damage through the release of pro-inflammatory cytokines and oxidative mediators (42). EOS% were also identified as a significant predictor of MGCAA. Eosinophils are involved in type 2 immunity, host defense, immune regulation, and tissue homeostasis (43). Autopsy studies have shown eosinophilic infiltration around the microvasculature in KD patients, suggesting a pathogenic role in vasculitis development (44).

Elevated CRP levels were positively correlated with MGCAA risk, consistent with the AHA statement that CRP reflects inflammatory severity and coronary risk in KD (6). While elevated NEU% have frequently been associated with increased CALs risk (45, 46), some studies have also suggested that lower NEU% may predispose patients to aneurysm formation (47). In our cohort, a lower NEU% was significantly associated with MGCAA, although the underlying mechanisms remain to be elucidated and warrant further investigation.

The absence of rash was associated with an increased risk of MGCAA, likely because missing this typical sign reflects an incomplete presentation of KD that can delay diagnosis and treatment, prolong inflammation, and increase the risk of coronary artery complications (48). Elevated TG were another significant predictor identified in our model. KD patients often exhibit hypertriglyceridemia, which is associated with a hypercoagulable state and potential vascular injury (49).

### 4.5 Calibration and clinical application

Discrimination and calibration are key metrics for evaluating prediction models with binary outcomes. In practice, a model’s discriminatory ability often remains relatively stable in new settings, whereas its calibration may be suboptimal and can deteriorate over time, a phenomenon known as calibration drift (50). As the validation dataset differs more from the development dataset in terms of case mix, outcome prevalence, predictor measurement and timing, or outcome definition, the likelihood of miscalibration correspondingly increases (26).Because the model showed miscalibration driven by differences in outcome incidence between cohorts (Suzhou 2.4% vs. Fuzhou 9.7%), we updated the model using an intercept-only recalibration (51).

In prediction model research, TRIPOD+AI and PROBAST+AI emphasize that model performance should be reported comprehensively, including discrimination, calibration, and DCA, to fully reflect the model’s real-world clinical value (21, 27). The DCA demonstrated that the model provided meaningful net clinical benefit across a range of threshold probabilities (internal validation: 0.01–0.28; external validation: 0.07–0.22). Within this range, decisions guided by the model yielded greater net benefit than either the “treat all” or “treat none” strategies. Notably, this clinically relevant threshold range corresponded well with the predicted risk distribution of most patients in both centers. When the threshold probability exceeded this range, the model’s net benefit gradually declined and approached that of the “treat none” strategy, indicating limited additional value at higher decision thresholds. Overall, the DCA results support the model’s potential for individualized risk stratification.

### 4.6 Limitations

This study has several limitations. First, although a multicenter design was employed, the retrospective nature of the study and the inclusion of participants from only two centers located in two provinces in eastern China may have introduced selection bias. Large-scale, prospective multicenter studies are needed to further validate the model’s generalizability across broader populations. Second, the relatively low prevalence of MGCAA in the cohorts may affect the stability and robustness of the predictive model. The model demonstrated moderate discrimination, indicating room for further improvement in predictive performance. Third, despite the use of a standardized coronary artery Z-score system, inter-operator variability in ultrasound techniques and image interpretation may have introduced measurement bias. Fourth, the lack of longitudinal follow-up data limits the assessment of long-term clinical outcomes and prevents further evaluation of potential calibration drift over time. Emerging or non-routine biomarkers were not incorporated into the model but may have potential research value. For example, immunological parameters such as double-negative T (DNT) cells and inflammatory cytokines such as interleukin-1 (IL-1) may be considered in future studies to further optimize model performance. Fifth, substantial inter-center heterogeneity in MGCAA incidence between the Suzhou and Fuzhou cohorts was observed, but insufficient data precluded exploration of its causes, limiting assessment of calibration differences and model transportability.

## 5 Conclusion

This study developed an interpretable machine learning model for predicting the risk of MGCAA in children with KD using seven routinely available predictors (time to diagnosis, MONO%, rash, EOS%, CRP, TG, and NEU%). During external validation, intercept-only recalibration was performed to address miscalibration arising from differences in outcome incidence between cohorts. DCA demonstrated meaningful net clinical benefit across clinically relevant threshold probabilities, supporting the model’s value for risk stratification. Further large-scale, prospective, and geographically diverse validations are warranted to confirm the model’s generalizability.

## Data Availability

The raw data supporting the conclusions of this article will be made available by the authors, without undue reservation.
