# Supplementary material for: Development and validation of an interpretable machine learning model for predicting medium-to-giant coronary aneurysms in Kawasaki disease
Source: Front Immunol. 2026 Mar 24;17:1804276. doi: 10.3389/fimmu.2026.1804276 (PMC13053267; doi:10.3389/fimmu.2026.1804276)
Supplement: Supplementary file 1 [file DataSheet1.doc]

**Supplementary Materials**

**Study Design and Population**

This retrospective study included pediatric Kawasaki disease (KD) patients from two tertiary hospitals in China. The development cohort was drawn from the Children’s Hospital of Soochow University (Mar 1, 2019–Jun 30, 2024), and the external validation cohort from Fujian Provincial Hospital (May 1, 2012–May 31, 2024). KD was diagnosed according to the American Heart Association (AHA) criteria. Classic KD is defined as persistent fever for ≥5 days accompanied by at least four of the following principal features: mucosal changes, conjunctivitis, polymorphous rash, extremity changes, and cervical lymphadenopathy(1, 2). When subjective interpretation of predictors was required, assessments were independently performed by two physicians with over five years of clinical experience. Inclusion criteria were: (1) a confirmed diagnosis of KD; and (2) echocardiographic assessment completed before IVIG treatment. Exclusion criteria were: (1) incomplete echocardiographic data; (2) IVIG treatment administered prior to hospital admission; (3) corticosteroid therapy before or during initial IVIG administration; and (4) a history of recurrent KD. Coronary artery measurements were performed by two pediatric cardiologists with over 10 years of echocardiography experience using standardized protocols. Echocardiographic assessments were conducted on GE Vivid E90, Vivid E95, Vivid 7, and Philips EPIQ 7C, EPIQ CV, and iE33 systems to ensure measurement consistency and reproducibility. Z-scores were calculated using the Dallaire method (3), and coronary artery involvement was classified as follows: no dilatation (Z < 2.0), mild dilatation (Z = 2.0 to < 2.5), small aneurysm (Z ≥ 2.5 to < 5.0), medium aneurysm (Z ≥ 5.0 to 10.0, diameter < 8 mm), giant aneurysm (Z ≥ 10.0 or diameter ≥ 8 mm) (1). Patients with medium or giant coronary artery aneurysms were defined as the positive outcome group.

**Data Collection and Preprocessing**

Clinical and laboratory data were extracted from electronic medical records (EMRs), and all candidate predictors were measured prior to intravenous immunoglobulin (IVIG) administration. To ensure data accuracy, two independent investigators randomly re-checked 20% of the variables. Variables available at only one center were excluded (Supplementary Table 2).

The Suzhou cohort was split into a training set (70%) and an internal validation set (30%) with a fixed random seed (set.seed(123)). Missing data were handled using multiple imputation by chained equations (MICE), with imputation performed separately in the training and test sets to avoid data leakage(4). Predictive mean matching was applied for continuous variables, logistic regression for binary variables, and polytomous regression for multicategorical variables. A total of 10 imputed datasets (m = 10) were generated with 20 iterations per imputation.

Spearman correlation analysis was performed to assess multicollinearity, and variable pairs with correlation coefficients greater than 0.6 were evaluated for redundancy. The final set of included variables is presented in Table 1.

**Machine Learning Model Development**

Eleven ML algorithms were developed, including Gradient Boosting Machine (GBM), Support Vector Machine (SVM), Random Forest (RF), K-Nearest Neighbors (KNN), Generalized Linear Model via Elastic Net (GLMnet), Decision Tree (DT), SVM with kernlab package (SVM kernlab), RF with ranger implementation (RF ranger), Adaptive Boosting (Adaboost), Naïve Bayes (NB), and Neural Network (NNET). All data preprocessing, including class balancing, hyperparameter tuning, and cross-validation, was performed exclusively within the training set to avoid data leakage.

Given the extremely low prevalence of medium-to-giant coronary artery aneurysms (MGCAA) in the development cohort (55/2334, 2.4%), ROSE (Random Over-Sampling Examples; R package ROSE) was applied only in the training set to generate synthetic minority samples for class balancing, while the internal and external validation datasets were kept untouched to preserve the true outcome prevalence for unbiased evaluation(5).

Model training was conducted using the *caret* package in R. We applied repeated 10-fold cross-validation (method = “repeatedcv”, number = 10, repeats = 5) within the training set, and model performance was optimized using the area under the receiver operating characteristic curve (AUC) (metric = “ROC”). Performance metrics included AUC, sensitivity, specificity, accuracy, F1 score, positive predictive value (PPV), and negative predictive value (NPV).The final model selection was based on the highest mean AUC across all resampling folds.

**Feature Selection and Model Interpretation**

Feature importance and interpretability were evaluated using SHapley Additive exPlanations(SHAP), and recursive feature elimination (RFE) was applied to identify the optimal subset of predictors(6).

**Recalibration and Clinical Utility Assessment**

Because ROSE oversampling in the training set altered the effective event prevalence, we prespecified a recalibration step to correct potential miscalibration when applying the model to data with the original outcome distribution. An intercept-only recalibration approach was used logit(p_new) = α + 1 × logit(p_pred). Where p_pred is the predicted probability from the original model and α is the intercept estimated from the internal validation data. Model calibration before and after recalibration was evaluated using the observed-to-expected (O/E) ratio, calibration slope, and Brier score. Decision curve analysis (DCA) was performed to evaluate the clinical usefulness of the model across a range of threshold probabilities.

**Web-Based Tool Deployment**.

To facilitate clinical use, the final model was deployed as an interactive online platform using the R Shiny framework. This tool enables clinicians to input key clinical variables, which the system then uses to provide a real-time, individualized risk estimate for MGCAA. The optimal decision threshold was determined using the Youden index to assist clinical risk stratification.

**External Validation**

Before external validation, the minimum required sample size was estimated using the pmvalsampsize package in R, based on the event rate of the positive outcome, AUC, O/E ratio, and calibration slope, along with their 95% confidence intervals (CI)(7).

An intercept-only recalibration was performed in the external dataset. The original model’s predicted logit risk was retained as an offset, and the intercept (α) was re-estimated as: logit(p_new) = α + 1·(predicted logit-risk).Calibration performance before and after recalibration was evaluated using the O/E ratio, calibration slope, and Brier score, while discrimination was assessed using the AUC.

**Quality, Risk of Bias, and Applicability Assessment**

This study used the PROBAST+AI (Prediction model Risk Of Bias ASsessment Tool for Artificial Intelligence), based on a domain-structured framework of signalling questions, to systematically assess the methodological quality, risk of bias, and applicability of the included prediction models in both the model development and model evaluation phases(8). The model development phase was assessed across four domains (participants and data sources, predictors, outcomes, and analysis) using 16 signalling questions, while the model evaluation phase was assessed across the same four domains using 18 signalling questions. Each signalling question was rated as Yes (Y), Probably Yes (PY), Probably No (PN), No (N), No Information (NI), or Not Applicable (NA). A domain was judged as low risk when all signalling questions were rated Y or PY; high risk when any was rated PN or N; otherwise, it was judged as unclear. Applicability was assessed in both phases based on the first three domains using the same rules. Overall judgement was defined as low risk when all domains were low risk, high risk when any domain was high risk, and unclear otherwise.

**Supplementary Tables**

Supplementary Table 1. TRIPOD+AI checklist for reporting the development and evaluation of the prediction model.

| **Section/Topic Item** | | **Development**  **/ evaluation**^1^ | **Checklist item** | **Reported**  **on page** |
| --- | --- | --- | --- | --- |
| **TITLE** | | | |  |
| *Title* | 1 | D;E | Identify the study as developing or evaluating the performance of a multivariable prediction model, the target population, and the outcome to be predicted | Page 1 |
| **ABSTRACT** | | | |  |
| *Abstract* | 2 | D;E | See TRIPOD+AI for Abstracts checklist | Page 1 |
| **INTRODUCTION** | | | |  |
| *Background* | 3a | D;E | Explain the healthcare context (including whether diagnostic or prognostic) and rationale for developing or evaluating the prediction model, including references to existing models | Page 2 |
|  | 3b | D;E | Describe the target population and the intended purpose of the prediction model in the context of the care pathway, including its intended users (e.g., healthcare professionals, patients, public) | Page 2 |
|  | 3c | D;E | Describe any known health inequalities between sociodemographic groups | Not reported |
| *Objectives* | 4 | D;E | Specify the study objectives, including whether the study describes the development or validation of a prediction model (or both) | Page 2 |
| **METHODS** | | | |  |
| *Data* | 5a | D;E | Describe the sources of data separately for the development and evaluation datasets (e.g., randomised trial, cohort, routine care or registry data), the rationale for using these data, and representativeness of the data | Page 2 |
|  | 5b | D;E | Specify the dates of the collected participant data, including start and end of participant accrual; and, if applicable, end of follow-up | Page 2 |
| *Participants* | 6a | D;E | Specify key elements of the study setting (e.g., primary care, secondary care, general population) including the number and location of centres | Page 2 |
|  | 6b | D;E | Describe the eligibility criteria for study participants | Supplementary Materials |
|  | 6c | D;E | Give details of any treatments received, and how they were handled during model development or evaluation, if relevant | Page 2 |
| *Data preparation* | 7 | D;E | Describe any data pre-processing and quality checking, including whether this was similar across relevant sociodemographic groups | Page 2–3 |
| *Outcome* | 8a | D;E | Clearly define the outcome that is being predicted and the time horizon, including how and when  assessed, the rationale for choosing this outcome, and whether the method of outcome assessment is consistent across sociodemographic groups | Supplementary Materials |
|  | 8b | D;E | If outcome assessment requires subjective interpretation, describe the qualifications and demographic characteristics of the outcome assessors | Supplementary Materials |
|  | 8c | D;E | Report any actions to blind assessment of the outcome to be predicted | Not reported |
| *Predictors* | 9a | D | Describe the choice of initial predictors (e.g., literature, previous models, all available predictors) and any pre-selection of predictors before model building | Supplementary Materials |
|  | 9b | D;E | Clearly define all predictors, including how and when they were measured (and any actions to blind assessment of predictors for the outcome and other predictors) | Page 3 |
|  | 9c | D;E | If predictor measurement requires subjective interpretation, describe the qualifications and demographic characteristics of the predictor assessors | Supplementary Materials |
| *Sample size* | 10 | D;E | Explain how the study size was arrived at (separately for development and evaluation), and justify that the study size was sufficient to answer the research question. Include details of any sample size  calculation | Page 3, 9 |
| *Missing data* | 11 | D;E | Describe how missing data were handled. Provide reasons for omitting any data | Page 3, 6 |
| *Analytical methods* | 12a | D | Describe how the data were used (e.g., for development and evaluation of model performance) in the analysis, including whether the data were partitioned, considering any sample size requirements | Page 2-5 |
|  | 12b | D | Depending on the type of model, describe how predictors were handled in the analyses (functional form, rescaling, transformation, or any standardisation). | Page 2–3 |
|  | 12c | D | Specify the type of model, rationale^2^, all model-building steps, including any hyperparameter tuning, and method for internal validation | Page 2, 5 |
|  | 12d | D;E | Describe if and how any heterogeneity in estimates of model parameter values and model performance was handled and quantified across clusters (e.g., hospitals, countries). See TRIPOD-Cluster for  additional considerations^3^ | Page 3, 11 |
|  | 12e | D;E | Specify all measures and plots used (and their rationale) to evaluate model performance (e.g., discrimination, calibration, clinical utility) and, if relevant, to compare multiple models | Page 3-7 |
|  | 12f | E | Describe any model updating (e.g., recalibration) arising from the model evaluation, either overall or for particular sociodemographic groups or settings | Page 3, 6, 7 |
|  | 12g | E | For model evaluation, describe how the model predictions were calculated (e.g., formula, code, object, application programming interface) | Page 3, 6, 10 |
| *Class imbalance* | 13 | D;E | If class imbalance methods were used, state why and how this was done, and any subsequent methods to recalibrate the model or the model predictions | Page 2-3, 6-7 |
| *Fairness* | 14 | D;E | Describe any approaches that were used to address model fairness and their rationale | Not reported |
| *Model output* | 15 | D | Specify the output of the prediction model (e.g., probabilities, classification). Provide details and rationale for any classification and how the thresholds were identified | Page 3-6, 10 |
| *Training versus evaluation* | 16 | D;E | Identify any differences between the development and evaluation data in healthcare setting, eligibility criteria, outcome, and predictors | Page 2-3 |
| *Ethical approval* | 17 | D;E | Name the institutional research board or ethics committee that approved the study and describe the participant-informed consent or the ethics committee waiver of informed consent | Page 2, 12 |
| **OPEN SCIENCE** | | | | |
| *Funding* | 18a | D;E | Give the source of funding and the role of the funders for the present study | Page 13 |
| *Conflicts of interest* | 18b | D;E | Declare any conflicts of interest and financial disclosures for all authors | Page 13 |
| *Protocol* | 18c | D;E | Indicate where the study protocol can be accessed or state that a protocol was not prepared | Not reported |
| *Registration* | 18d | D;E | Provide registration information for the study, including register name and registration number, or state that the study was not registered | Not reported |
| *Data sharing* | 18e | D;E | Provide details of the availability of the study data | Page 13 |
| *Code sharing* | 18f | D;E | Provide details of the availability of the analytical code^4^ | Not reported |
| **PATIENT & PUBLIC INVOLVEMENT** | | | | |
| *Patient & Public Involvement* | 19 | D;E | Provide details of any patient and public involvement during the design, conduct, reporting,  interpretation, or dissemination of the study or state no involvement. | Not reported |
| **RESULTS** | | | | |
| *Participants* | 20a | D;E | Describe the flow of participants through the study, including the number of participants with and  without the outcome and, if applicable, a summary of the follow-up time. A diagram may be helpful. | Figure1 |
|  | 20b | D;E | Report the characteristics overall and, where applicable, for each data source or setting, including the key dates, key predictors (including demographics), treatments received, sample size, number of  outcome events, follow-up time, and amount of missing data. A table may be helpful. Report any differences across key demographic groups. | Baseline table |
|  | 20c | E | For model evaluation, show a comparison with the development data of the distribution of important predictors (demographics, predictors, and outcome). | Supplementary Table 5 |
| *Model development* | 21 | D;E | Specify the number of participants and outcome events in each analysis (e.g., for model development, hyperparameter tuning, model evaluation) | Page 2, 4-5 |
| *Model*  *specification* | 22 | D | Provide details of the full prediction model (e.g., formula, code, object, application programming interface) to allow predictions in new individuals and to enable third-party evaluation and  implementation, including any restrictions to access or re-use (e.g., freely available, proprietary)^5^ | Page 5, 6, 10 |
| *Model*  *performance* | 23a | D;E | Report model performance estimates with confidence intervals, including for any key subgroups (e.g., sociodemographic). Consider plots to aid presentation. | Page 4-8 |
|  | 23b | D;E | If examined, report results of any heterogeneity in model performance across clusters. See TRIPOD Cluster for additional details^3^. | Page 3, 7-9 |
| *Model updating* | 24 | E | Report the results from any model updating, including the updated model and subsequent performance | Page 3, 6-7 |
| **DISCUSSION** | | | | |
| *Interpretation* | 25 | D;E | Give an overall interpretation of the main results, including issues of fairness in the context of the objectives and previous studies | Page 9-11 |
| *Limitations* | 26 | D;E | Discuss any limitations of the study (such as a non-representative sample, sample size, overfitting, missing data) and their effects on any biases, statistical uncertainty, and generalizability | Page 12 |
| *Usability of the*  *model in the*  *context of current care* | 27a | D | Describe how poor quality or unavailable input data (e.g., predictor values) should be assessed and handled when implementing the prediction model | Page 6 |
|  | 27b | D | Specify whether users will be required to interact in the handling of the input data or use of the model, and what level of expertise is required of users | Page 3, 6 |
|  | 27c | D;E | Discuss any next steps for future research, with a specific view to applicability and generalizability of the model | Page 12 |

Notes: D=items relevant only to the development of a prediction model; E=items relating solely to the evaluation of a prediction model; D; E=items applicable to both the development and evaluation of a prediction model.

Supplementary Table 2. Variables available in only one center before harmonization.

| Fuzhou-only variables | Suzhou-only variables |
| --- | --- |
| BMI | AG |
| Breastfeeding_Duration | BASO% |
| MP_IgM | C3 |
| NT_proBNP | C4 |
| P | DBIL |
| TBIL | GLB |
|  | IBIL |
|  | IgA |
|  | IgG |
|  | IgM |
|  | LAC |
|  | LPS |
|  | PCT |
|  | RBC |
|  | URBC |
|  | Urine_Bacteria |
|  | cTnT |
|  | PH |

Abbreviations: BMI, body mass index; MP_IgM, Mycoplasma pneumoniae IgM; NT_proBNP, N-terminal pro-B-type natriuretic peptide; P, serum phosphorus; TBIL, total bilirubin; AG, anion gap; BASO%, basophil percentage; C3, complement 3; C4, complement 4; DBIL, direct bilirubin; GLB, globulin; IBIL, indirect bilirubin; IgA, immunoglobulin A; IgG, immunoglobulin G; IgM, immunoglobulin M; LAC, lactate; LPS, lipopolysaccharide; PCT, procalcitonin; RBC, red blood cell; URBC, urine red blood cell; Urine_Bacteria, urinary bacteria; cTnT, cardiac troponin T; PH, blood pH.

Supplementary Table 3. Proportion of missing data for each variable in training and test sets.

| Variable | Training set | Percentage of missing data, % | Test set | Percentage of missing data, % |
| --- | --- | --- | --- | --- |
| Gender | 0 | 0 | 55 | 7.87 |
| Age at presentation | 0 | 0 | 9 | 1.29 |
| Time to diagnosis | 130 | 7.95 | 0 | 0 |
| Conjunctival injection | 0 | 0 | 0 | 0 |
| Oral mucosal change | 65 | 3.98 | 0 | 0 |
| Edema of the hands and feet | 0 | 0 | 0 | 0 |
| Rash | 0 | 0 | 0 | 0 |
| Cervical lymphadenopathy | 0 | 0 | 25 | 3.58 |
| Perianal desquamation | 0 | 0 | 27 | 3.86 |
| WBC | 118 | 7.22 | 0 | 0 |
| NEU% | 92 | 5.63 | 0 | 0 |
| EOS% | 0 | 0 | 0 | 0 |
| MONO% | 0 | 0 | 0 | 0 |
| PLT | 130 | 7.95 | 0 | 0 |
| Hb | 130 | 7.95 | 0 | 0 |
| LYMPH% | 0 | 0 | 0 | 0 |
| CRP | 0 | 0 | 0 | 0 |
| TG | 17 | 1.04 | 3 | 0.43 |
| TC | 17 | 1.04 | 55 | 7.87 |
| LDH | 3 | 0.18 | 41 | 5.87 |
| GLU | 0 | 0 | 14 | 2.00 |
| ALB | 130 | 7.95 | 0 | 0 |
| Ca | 0 | 0 | 55 | 7.87 |
| CK | 0 | 0 | 0 | 0 |
| AST | 0 | 0 | 0 | 0 |
| ALT | 0 | 0 | 0 | 0 |
| Cr | 0 | 0 | 0 | 0 |
| GGT | 109 | 6.67 | 55 | 7.87 |
| Mg | 0 | 0 | 55 | 7.87 |
| Na | 81 | 4.95 | 55 | 7.87 |
| K | 0 | 0 | 55 | 7.87 |
| Cl | 0 | 0 | 0 | 0 |
| ALP | 0 | 0 | 0 | 0 |
| BUN | 130 | 7.95 | 1 | 0.14 |
| CKMB | 0 | 0 | 0 | 0 |
| UWBC | 0 | 0 | 0 | 0 |

Abbreviations: WBC, white blood cell; NEU%, neutrophil percentage; EOS%, eosinophil percentage; MONO%, monocyte percentage; PLT, platelet count; Hb, hemoglobin; LYMPH%, lymphocyte percentage; CRP, C-reactive protein; TG, triglyceride; TC, total cholesterol; LDH, lactate dehydrogenase; GLU, glucose; ALB, serum albumin; CK, creatine kinase; AST, aspartate aminotransferase; ALT, alanine aminotransferase; Cr, creatinine; GGT, γ-glutamyl transpeptidase; ALP, alkaline phosphatase; BUN, blood urea nitrogen; CKMB, creatine kinase MB; UWBC, urinary white blood cell.

Supplementary Table 4. Comparison of clinical characteristics between patients with and without MGCAA in the fuzhou cohort.

| Characteristics | Non-MGCAA (n = 400) | MGCAA (n = 43) | *P* value |
| --- | --- | --- | --- |
| Male, n (%) | 272 (68.0) | 31 (72.1) | .583 |
| Age at presentation, months | 24.00 (15.00-48.00) | 24.00 (12.00-48.00) | .250 |
| Time to diagnosis, days | 6.00 (5.00-8.00) | 8.00 (6.00-11.00) | .001* |
| Conjunctival injection, n (%) | 349 (87.3) | 33 (76.7) | .057 |
| Oral mucosal change, n (%) | 358 (89.5) | 33 (76.7) | .014* |
| Edema of the hands and feet, n (%) | 162 (40.5) | 13 (30.2) | .191 |
| Rash, n (%) | 311 (77.8) | 27 (62.8) | .028* |
| Cervical lymphadenopathy, n (%) | 325 (81.3) | 29 (67.4) | .028* |
| Perianal desquamation, n (%) | 91 (22.8) | 8 (18.6) | .535 |
| WBC, ×10^9^/L | 11.64 (8.30-16.01) | 13.61 (8.52-18.93) | .237 |
| NEU% | 56.00 (36.00-73.00) | 46.00 (26.00-62.00) | .032* |
| EOS% | 2.10 (0.73-4.60) | 2.40 (0.80-5.70) | .412 |
| MONO% | 6.30 (5.00-8.30) | 6.30 (5.10-7.50) | .547 |
| PLT, ×10^9^/L | 351.50 (270.00-457.75) | 404.00 (326.00-502.00) | .032* |
| Hb, g/L | 110.00 (103.00-119.00) | 107.00 (96.00-113.00) | .004* |
| LYMPH% | 32.50 (19.00-51.00) | 41.00 (27.00-60.00) | .022* |
| CRP, mg/L | 57.19 (29.37-88.17) | 51.60 (28.60-92.34) | .658 |
| TG, mmol/L | 1.23 (0.95-1.59) | 1.26 (0.98-1.83) | .249 |
| TC, mmol/L | 3.29 (2.90-3.75) | 3.27 (2.90-3.80) | .787 |
| LDH, U/L | 290.80 (247.00-401.00) | 311.70 (247.00-421.50) | .442 |
| GLU, mmol/L | 5.53 (4.88-6.22) | 5.39 (4.96-6.19) | .597 |
| ALB, g/L | 37.40 (33.90-40.00) | 36.30 (33.00-39.20) | .255 |
| Ca, mmol/L | 2.33 (2.22-2.41) | 2.31 (2.18-2.42) | .623 |
| CK, U/L | 48.00 (31.00-76.00) | 32.00 (22.00-65.00) | .030* |
| AST, U/L | 35.00 (26.85-54.00) | 38.20 (28.90-54.00) | .485 |
| ALT, U/L | 26.00 (14.00-69.08) | 23.30 (15.00-65.90) | .677 |
| Cr, μmol/L | 27.00 (20.00-36.00) | 29.00 (20.00-33.00) | .711 |
| GGT, U/L | 24.00 (13.00-80.75) | 31.00 (13.00-85.00) | .830 |
| Mg, mmol/L | 0.88 (0.84-0.93) | 0.89 (0.86-0.95) | .192 |
| Na, mmol/L | 135.00 (133.50-137.00) | 135.10 (133.60-137.80) | .727 |
| K, mmol/L | 4.38 (3.97-4.70) | 4.38 (4.10-4.90) | .304 |
| Cl, mmol/L | 99.75 (98.00-102.00) | 100.10 (98.30-102.10) | .639 |
| ALP, U/L | 179.90 (148.28-246.65) | 170.00 (151.40-237.00) | .713 |
| BUN, mmol/L | 2.80 (2.20-3.60) | 2.60 (1.80-3.40) | .131 |
| CKMB, ng/ml | 21.65 (14.00-32.00) | 21.00 (12.00-33.00) | .771 |
| UWBC, /μL | 0.00 (0.00-1.90) | 0.00 (0.00-0.10) | .299 |

Notes: Continuous variables are presented as median and interquartile range; categorical variables as numbers and percentages. * *P* < .05.

Abbreviations: MGCAA, medium-to-giant coronary artery aneurysms; WBC, white blood cell; NEU%, neutrophil percentage; EOS%, eosinophil percentage; MONO%, monocyte percentage; PLT, platelet count; Hb, hemoglobin; LYMPH%, lymphocyte percentage; CRP, C-reactive protein; TG, triglyceride; TC, total cholesterol; LDH, lactate dehydrogenase; GLU, glucose; ALB, serum albumin; CK, creatine kinase; AST, aspartate aminotransferase; ALT, alanine aminotransferase; Cr, creatinine; GGT, γ-glutamyl transpeptidase; ALP, alkaline phosphatase; BUN, blood urea nitrogen; CKMB, creatine kinase MB; UWBC, urinary white blood cell.

Supplementary Table 5. Comparison of clinical characteristics between the training and test cohorts, and between the suzhou and fuzhou cohorts.

| Characteristics | Training cohort  (n = 1635) | Test cohort  (n = 699) | *P* value | Suzhou cohort  (n = 2334) | Fuzhou cohort  (n = 443) | *P* value |
| --- | --- | --- | --- | --- | --- | --- |
| Male, n (%) | 995 (60.9) | 421 (60.2) | .776 | 1416 (60.7) | 303 (68.4) | .002^#^ |
| Age at presentation, months | 26.00 (14.00-46.00) | 27.00 (14.00-47.00) | .416 | 26.00 (14.00-47.00) | 24.00 (15.00-48.00) | .963 |
| Time to diagnosis, days | 6.00 (5.00-7.00) | 6.00 (5.00-7.00) | .404 | 6.00 (5.00-7.00) | 6.00 (5.00-8.00) | <.001^#^ |
| Conjunctival injection, n (%) | 192 (11.7) | 73 (10.4) | .365 | 2069 (88.6) | 382 (86.2) | .148 |
| Oral mucosal change, n (%) | 222 (13.6) | 83 (11.9) | .263 | 2029 (86.9) | 391 (88.3) | .443 |
| Edema of the hands and feet, n (%) | 883 (54.0) | 383 (54.8) | .727 | 1068 (45.8) | 175 (39.5) | .015^#^ |
| Rash, n (%) | 416 (25.4) | 172 (24.6) | .670 | 1746 (74.8) | 338 (76.3) | .506 |
| Cervical lymphadenopathy, n (%) | 189 (11.6) | 72 (10.3) | .377 | 2073 (88.8) | 354 (79.9) | <.001^#^ |
| Perianal desquamation, n (%) | 1491 (91.2) | 641 (91.7) | .688 | 202 (8.7) | 99 (22.3) | <.001^#^ |
| WBC, ×10^9^/L | 12.37 (8.69-16.34) | 12.36 (9.17-16.49) | .529 | 12.37 (8.88-16.35) | 11.82 (8.32-16.18) | .254 |
| NEU% | 59.70 (43.20-74.40) | 61.60 (45.40-75.20) | .091 | 60.30 (44.08-74.70) | 55.00 (36.00-73.00) | <.001^#^ |
| EOS% | 2.00 (0.60-4.00) | 2.00 (0.60-4.10) | .938 | 2.00 (0.60-4.00) | 2.10 (0.80-4.70) | .065 |
| MONO% | 6.40 (4.70-8.30) | 6.30 (4.60-8.50) | .851 | 6.40 (4.70-8.40) | 6.30 (5.00-8.10) | .431 |
| PLT, ×10^9^/L | 369.00 (287.00-463.00) | 361.00 (280.00-455.00) | .070 | 365.00 (285.00-461.00) | 357.00 (273.00-461.00) | .494 |
| Hb, g/L | 113.00 (107.00-120.00) | 113.00 (107.00-120.00) | .558 | 113.00 (107.00-120.00) | 109.00 (103.00-118.00) | <.001^#^ |
| LYMPH% | 29.90 (17.80-44.40) | 28.60 (17.20-41.60) | .073 | 29.50 (17.70-43.80) | 34.00 (19.00-53.00) | <.001^#^ |
| CRP, mg/L | 63.33 (36.41-99.32) | 67.06 (39.40-106.49) | .041* | 64.07 (37.45-101.17) | 56.76 (29.36-88.23) | <.001^#^ |
| TG, mmol/L | 1.14 (0.93-1.47) | 1.21 (0.92-1.52) | .141 | 1.16 (0.93-1.48) | 1.23 (0.95-1.60) | .022^#^ |
| TC, mmol/L | 3.65 (3.15-4.17) | 3.65 (3.15-4.10) | .672 | 3.65 (3.15-4.15) | 3.27 (2.90-3.75) | <.001^#^ |
| LDH, U/L | 356.00 (297.60-427.30) | 352.80 (294.90-427.10) | .664 | 354.80 (297.08-427.15) | 296.00 (247.00-402.00) | <.001^#^ |
| GLU, mmol/L | 5.40 (4.80-6.20) | 5.40 (4.80-6.10) | .907 | 5.40 (4.80-6.10) | 5.51 (4.88-6.20) | .103 |
| ALB, g/L | 40.00 (37.70-42.10) | 39.80 (37.40-41.80) | .141 | 40.00 (37.60-42.00) | 37.10 (33.90-40.00) | <.001^#^ |
| Ca, mmol/L | 2.19 (1.10-2.35) | 2.18 (1.10-2.33) | .441 | 2.19 (1.10-2.34) | 2.33 (2.22-2.41) | <.001^#^ |
| CK, U/L | 60.60 (41.60-87.80) | 58.20 (40.40-90.00) | .754 | 60.30 (41.40-88.33) | 48.00 (30.00-76.00) | <.001^#^ |
| AST, U/L | 33.80 (26.30-51.00) | 33.70 (26.40-48.80) | .561 | 33.80 (26.30-50.20) | 35.10 (27.00-54.00) | .065 |
| ALT, U/L | 22.10 (13.60-64.40) | 23.50 (13.50-70.80) | .503 | 22.30 (13.60-66.75) | 25.30 (14.00-69.00) | .141 |
| Cr, μmol/L | 24.60 (20.30-29.80) | 25.10 (20.60-30.90) | .114 | 24.70 (20.40-30.23) | 27.00 (20.00-36.00) | <.001^#^ |
| GGT, U/L | 19.60 (11.70-69.80) | 21.80 (11.70-74.70) | .469 | 20.20 (11.70-71.93) | 24.50 (13.00-81.00) | .109 |
| Mg, mmol/L | 0.99 (0.93-1.06) | 0.98 (0.92-1.05) | .072 | 0.99 (0.93-1.05) | 0.88 (0.84-0.93) | <.001^#^ |
| Na, mmol/L | 135.00 (133.00-137.00) | 135.00 (133.00-137.00) | .225 | 135.00 (133.00-137.00) | 135.00 (133.50-137.00) | .799 |
| K, mmol/L | 3.90 (3.60-4.30) | 3.90 (3.60-4.30) | .987 | 3.90 (3.60-4.30) | 4.38 (4.00-4.70) | <.001^#^ |
| Cl, mmol/L | 103.00 (101.00-105.00) | 103.00 (102.00-105.00) | .415 | 103.00 (102.00-105.00) | 99.80 (98.00-102.00) | <.001^#^ |
| ALP, U/L | 176.00 (147.00-223.00) | 175.00 (145.00-219.00) | .766 | 175.00 (146.75-222.00) | 179.40 (148.60-244.30) | .212 |
| BUN, mmol/L | 3.03 (2.38-3.76) | 3.09 (2.43-3.75) | .473 | 3.04 (2.40-3.76) | 2.80 (2.12-3.56) | <.001^#^ |
| CKMB, ng/ml | 1.20 (0.79-1.90) | 1.19 (0.73-1.80) | .196 | 1.20 (0.78-1.90) | 21.60 (14.00-32.00) | <.001^#^ |
| UWBC, /μL | 7.00 (2.10-22.00) | 8.00 (2.50-28.90) | .043* | 7.20 (2.30-23.30) | 0.00 (0.00-1.90) | <.001^#^ |

Notes: Continuous variables are presented as median and interquartile range; categorical variables as numbers and percentages. Suzhou cohort consists of the training cohort and the test cohort. * Comparison between the training cohort and the test cohort: *P* < .05; # Comparison between the Suzhou cohort and the Fuzhou cohort: *P* < .05.

Abbreviations: WBC, white blood cell; NEU%, neutrophil percentage; EOS%, eosinophil percentage; MONO%, monocyte percentage; PLT, platelet count; Hb, hemoglobin; LYMPH%, lymphocyte percentage; CRP, C-reactive protein; TG, triglyceride; TC, total cholesterol; LDH, lactate dehydrogenase; GLU, glucose; ALB, serum albumin; CK, creatine kinase; AST, aspartate aminotransferase; ALT, alanine aminotransferase; Cr, creatinine; GGT, γ-glutamyl transpeptidase; ALP, alkaline phosphatase; BUN, blood urea nitrogen; CKMB, creatine kinase MB; UWBC, urinary white blood cell.

Supplementary Table 6. Clinical characteristics by grade of coronary artery lesions in the suzhou and fuzhou regions.

| Characteristics | Non-MGCAA (n = 2679) | | | MGCAA (n = 98) | |
| --- | --- | --- | --- | --- | --- |
|  | No involvement  (n = 1915) | Dilation only  (n = 317) | Small CAA  (n = 447) | Medium CAA  (n = 88) | Large or giant CAA  (n = 10) |
| Male, n (%) | 1108 (57.9) | 222 (70.0) | 319 (71.4) | 67 (76.1) | 3 (30.0) |
| Age at presentation, months | 29.00 (16.00-48.00) | 22.00 (12.00-44.00) | 22.00 (12.00-36.00) | 20.00 (10.00-48.00) | 32.50 (23.25-41.75) |
| Time to diagnosis, days | 6.00 (5.00-7.00) | 5.00 (4.00-7.00) | 6.00 (5.00-7.00) | 8.00 (6.00-10.00) | 9.00 (5.00-13.25) |
| Conjunctival injection, n (%) | 1708 (89.2) | 273 (86.1) | 397 (88.8) | 66 (75.0) | 7 (70.0) |
| Oral mucosal change, n (%) | 1707 (89.1) | 261 (82.3) | 379 (84.8) | 64 (72.7) | 9 (90.0) |
| Edema of the hands and feet, n (%) | 898 (46.9) | 117 (36.9) | 194 (43.4) | 31 (35.2) | 3 (30.0) |
| Rash, n (%) | 1456 (76.0) | 232 (73.2) | 337 (75.4) | 50 (56.8) | 9 (90.0) |
| Cervical lymphadenopathy, n (%) | 1720 (89.8) | 266 (83.9) | 371 (83.0) | 63 (71.6) | 7 (70.0) |
| Perianal desquamation, n (%) | 210 (11.0) | 25 (7.9) | 53 (11.9) | 12 (13.6) | 1 (10.0) |
| WBC, ×10^9^/L | 12.05 (8.67-15.90) | 12.06 (8.21-16.99) | 12.76 (9.10-16.64) | 14.06 (9.46-19.49) | 16.12 (11.52-17.91) |
| NEU% | 60.70 (43.50-74.50) | 61.20 (41.10-76.50) | 56.60 (39.10-72.20) | 51.20 (33.13-65.23) | 47.55 (39.18-67.25) |
| EOS% | 1.90 (0.60-3.90) | 2.00 (0.65-4.00) | 2.30 (0.70-5.00) | 2.55 (0.80-5.15) | 1.85 (0.63-4.53) |
| MONO% | 6.30 (4.60-8.30) | 6.60 (4.85-8.65) | 6.40 (5.00-8.50) | 6.70 (4.90-8.58) | 5.95 (4.28-8.25) |
| PLT, ×10^9^/L | 360.00 (281.00-453.00) | 358.00 (279.50-471.00) | 385.00 (284.00-484.00) | 416.50 (330.00-573.00) | 433.00 (328.50-482.75) |
| Hb, g/L | 114.00 (107.00-120.00) | 112.00 (105.00-119.00) | 111.00 (104.00-119.00) | 108.00 (98.25-113.75) | 111.00 (99.50-119.00) |
| LYMPH% | 29.30 (17.80-44.00) | 29.30 (16.30-45.65) | 32.00 (19.00-48.90) | 36.25 (22.20-55.98) | 38.55 (23.35-49.50) |
| CRP, mg/L | 61.01 (34.29-96.19) | 72.28 (39.22-112.99) | 63.76 (39.33-102.89) | 56.55 (31.27-103.65) | 101.65 (38.55-146.25) |
| TG, mmol/L | 1.14 (0.91-1.48) | 1.20 (0.94-1.55) | 1.24 (0.98-1.57) | 1.26 (1.00-1.63) | 1.14 (0.98-1.89) |
| TC, mmol/L | 3.63 (3.15-4.14) | 3.43 (3.01-3.93) | 3.48 (3.00-3.94) | 3.40 (3.06-4.12) | 4.08 (2.73-4.44) |
| LDH, U/L | 346.00 (286.60-424.50) | 357.50 (290.50-419.40) | 344.60 (286.20-426.80) | 333.50 (265.38-466.75) | 348.25 (302.38-416.43) |
| GLU, mmol/L | 5.47 (4.80-6.20) | 5.40 (4.85-6.10) | 5.40 (4.90-6.20) | 5.38 (4.73-5.90) | 5.16 (4.98-5.63) |
| ALB, g/L | 39.80 (37.30-41.90) | 39.70 (36.90-41.95) | 39.00 (36.00-41.30) | 37.55 (34.93-40.53) | 39.35 (32.98-41.60) |
| Ca, mmol/L | 2.22 (1.11-2.36) | 2.16 (1.11-2.35) | 2.26 (1.24-2.38) | 2.26 (2.06-2.37) | 2.18 (1.13-2.28) |
| CK, U/L | 62.00 (41.60-88.00) | 53.80 (40.15-83.80) | 53.50 (34.70-82.20) | 40.70 (26.85-62.40) | 52.70 (22.90-83.95) |
| AST, U/L | 33.80 (26.00-51.30) | 35.90 (28.05-54.00) | 34.00 (26.50-48.90) | 33.00 (26.23-51.08) | 40.35 (28.10-59.83) |
| ALT, U/L | 21.00 (13.00-65.70) | 27.90 (14.85-80.85) | 26.00 (16.20-68.10) | 20.40 (14.05-60.68) | 25.75 (18.33-48.23) |
| Cr, μmol/L | 25.10 (20.50-30.90) | 24.80 (20.80-31.05) | 24.30 (20.00-30.70) | 25.00 (18.13-30.58) | 27.40 (21.73-35.95) |
| GGT, U/L | 18.50 (11.30-66.90) | 22.60 (12.65-82.70) | 29.00 (13.80-85.10) | 28.35 (14.00-76.30) | 42.50 (15.53-91.38) |
| Mg, mmol/L | 0.97 (0.91-1.04) | 0.97 (0.91-1.05) | 0.96 (0.89-1.04) | 0.95 (0.88-1.01) | 0.95 (0.88-1.04) |
| Na, mmol/L | 135.00 (133.00-137.00) | 135.00 (133.00-137.00) | 135.00 (133.00-137.00) | 136.00 (134.00-137.95) | 137.50 (133.75-140.00) |
| K, mmol/L | 4.00 (3.61-4.30) | 4.00 (3.68-4.30) | 4.10 (3.70-4.40) | 4.20 (3.80-4.59) | 3.95 (3.79-4.23) |
| Cl, mmol/L | 103.00 (101.00-105.00) | 103.00 (101.00-105.00) | 103.00 (100.00-105.00) | 101.45 (99.00-104.00) | 102.50 (100.10-105.50) |
| ALP, U/L | 175.00 (147.00-222.00) | 182.00 (149.50-231.50) | 177.00 (144.00-228.00) | 170.00 (146.83-218.75) | 208.50 (145.75-246.25) |
| BUN, mmol/L | 3.03 (2.36-3.78) | 3.08 (2.44-3.64) | 2.88 (2.30-3.61) | 2.80 (2.13-3.64) | 3.63 (2.12-4.40) |
| CKMB, ng/ml | 1.30 (0.80-2.70) | 1.40 (0.88-2.30) | 1.40 (0.82-3.20) | 2.55 (0.93-20.20) | 2.15 (0.60-19.70) |
| UWBC, /μL | 5.40 (1.20-20.00) | 6.00 (1.60-22.45) | 6.00 (1.00-17.00) | 1.05 (0.00-4.88) | 7.20 (0.08-15.63) |

Notes: Continuous variables are presented as median and interquartile range; categorical variables as numbers and percentages.

Abbreviations: MGCAA, medium-to-giant coronary artery aneurysms; WBC, white blood cell; NEU%, neutrophil percentage; EOS%, eosinophil percentage; MONO%, monocyte percentage; PLT, platelet count; Hb, hemoglobin; LYMPH%, lymphocyte percentage; CRP, C-reactive protein; TG, triglyceride; TC, total cholesterol; LDH, lactate dehydrogenase; GLU, glucose; ALB, serum albumin; CK, creatine kinase; AST, aspartate aminotransferase; ALT, alanine aminotransferase; Cr, creatinine; GGT, γ-glutamyl transpeptidase; ALP, alkaline phosphatase; BUN, blood urea nitrogen; CKMB, creatine kinase MB; UWBC, urinary white blood cell.

# Supplementary Table 7. MGCAA class distribution before and after ROSE across training, test, and external validation sets.

| Sampling stage | Non-MGCAA (n) | Non-MGCAA (%) | MGCAA (n) | MGCAA (%) | Total (n) |
| --- | --- | --- | --- | --- | --- |
| Before ROSE (Training set) | 1596 | 97.61 | 39 | 2.39 | 1635 |
| After ROSE (Training set) | 833 | 50.95 | 802 | 49.05 | 1635 |
| Test set | NA | NA | NA | NA | NA |
| External validation set | NA | NA | NA | NA | NA |

Abbreviations: MGCAA, medium-to-giant coronary artery aneurysms; ROSE, Random Over-Sampling Examples; NA, not applicable.

Supplementary Table 8. Predictive performance of the SVM kernlab and RF ranger models for MGCAA with different numbers of features.

| Model | Number of features | AUC | Sensitivity | Specificity | PPV | NPV |
| --- | --- | --- | --- | --- | --- | --- |
| **SVM kernlab** | | | | | | |
|  | 32 | 0.733 | 0.688 | 0.775 | 0.991 | 0.267 |
|  | 27 | 0.741 | 0.688 | 0.758 | 0.990 | 0.263 |
|  | 22 | 0.715 | 0.563 | 0.747 | 0.986 | 0.249 |
|  | 17 | 0.732 | 0.688 | 0.728 | 0.990 | 0.256 |
|  | 12 | 0.697 | 0.625 | 0.728 | 0.988 | 0.251 |
|  | 7 | 0.732 | 0.688 | 0.703 | 0.990 | 0.251 |
|  | 2 | 0.699 | 0.625 | 0.761 | 0.989 | 0.258 |
| **RF ranger** | | | | | | |
|  | 32 | 0.765 | 0.688 | 0.713 | 0.990 | 0.153 |
|  | 27 | 0.773 | 0.688 | 0.726 | 0.990 | 0.156 |
|  | 22 | 0.770 | 0.688 | 0.813 | 0.991 | 0.179 |
|  | 17 | 0.740 | 0.688 | 0.804 | 0.991 | 0.176 |
|  | 12 | 0.671 | 0.375 | 0.833 | 0.885 | 0.215 |
|  | 7 | 0.694 | 0.563 | 0.805 | 0.889 | 0.222 |
|  | 2 | 0.699 | 0.625 | 0.774 | 0.890 | 0.204 |

Abbreviations: MGCAA, medium-to-giant coronary artery aneurysms; AUC, area under the receiver operating characteristic curve; PPV, positive predictive value; NPV, negative predictive value; SVM kernlab, support vector machine with kernlab algorithm; RF ranger, random forest with ranger algorithm.

Supplementary Table 9. PROBAST+AI assessment of methodological quality, risk of bias, and applicability of the prediction model.

| **Model development:Signalling questions** | **Assessment**  **(Y/PY/PN/N/NI/NA)** | **Model evaluation:Signalling questions** | **Assessment**  **(Y/PY/PN/N/NI/NA)** |
| --- | --- | --- | --- |
| **Participants and data sources** | | | |
| 1.1 Were appropriate data sources used? | Y | 1.1 Were appropriate data sources used? | Y |
| 1.2 Was an appropriate study design used? | N | 1.2 Was an appropriate study design used? | N |
| 1.3 Did the inclusions and exclusions of study participants result in a representative dataset? | Y | 1.3 Did the inclusions and exclusions of study participants result in a representative dataset? | Y |
| Quality: Concern regarding quality of selection of participants and data sources | High Risk | Quality: Concern regarding quality of selection of participants and data sources | High Risk |
| Applicability: Concern that the data of the included participants do not match the review question or the assessor’s intended use of the prediction model | Low Risk | Applicability: Concern that the data of the included participants do not match the review question or the assessor’s intended use of the prediction model | Low Risk |
| **Predictors** | | | |
| 2.1 Were predictors defined and assessed in a similar way for all participants? | Y | 2.1 Were predictors defined and assessed in a similar way for all participants? | Y |
| 2.2 Was any preprocessing of predictors similar for all participants? | Y | 2.2 Was any preprocessing of predictors similar for all participants? | Y |
| 2.3 Were predictor assessments made without knowledge of outcome data? | N | 2.3 Were predictor assessments made without knowledge of outcome data? | N |
| 2.4 Were the predictors included in the model available at the time the model was intended to be used? | Y | 2.4 Were the predictors included in the model available at the time the model was intended to be used? | Y |
| Quality: Concern regarding the quality of the predictors or their assessment | High Risk | Quality: Concern regarding the quality of the predictors or their assessment | High Risk |
| Applicability: Concern that the definition, preprocessing, assessment, or timing of assessment of the predictors in the model do not match the review question or the assessor’s intended use | Low Risk | Applicability: Concern that the definition, preprocessing, assessment, or timing of assessment of the predictors in the model do not match the review question or the assessor’s intended use | Low Risk |
| **Outcomes** | |  |  |
| 3.1 Were outcomes defined and assessed appropriately? | Y | 3.1 Were outcomes defined and assessed appropriately? | Y |
| 3.2 Were outcomes defined and assessed in a similar way for all participants? | Y | 3.2 Were outcomes defined and assessed in a similar way for all participants? | Y |
| 3.3 Were outcome assessments made without use or knowledge of predictor data? | Y | 3.3 Were outcome assessments made without use or knowledge of predictor data? | Y |
| 3.4 Was the time interval between predictor assessment and outcome assessment appropriate? | Y | 3.4 Was the time interval between predictor assessment and outcome assessment appropriate? | Y |
| Quality: Concern regarding quality of the outcome or its determination | Low Risk | Quality: Concern regarding quality of the outcome or its determination | Low Risk |
| Applicability: Concern that the outcome, its definition, assessment, or timing of assessment do not match the review question or the assessor’s intended use | Low Risk | Applicability: Concern that the outcome, its definition, assessment, or timing of assessment do not match the review question or the assessor’s intended use | Low Risk |
| **Analyses** | | | |
| 4.1 Was there evidence that the sample size was reasonable? | Y | 4.1 Was model evaluation based on only apparent performance avoided? | Y |
| 4.2 Were continuous and categorical predictors handled appropriately? | Y | 4.2 Was there evidence that the sample size was reasonable? | Y |
| 4.3 Were participants with missing or censored data handled appropriately in the analysis? | Y | 4.3 Were participants with missing or censored data handled appropriately in the analysis? | Y |
| 4.4 If methods to address class imbalance were used, was the model or the model predictions recalibrated? | Y | 4.4 If methods to address class imbalance were used, was the evaluation done in a dataset without correction for imbalance? | Y |
| 4.5 Were methods used to address potential model overfitting? | Y | 4.5 If data splitting was done to create training and test datasets, was there evidence that data leakage was avoided? | Y |
|  |  | 4.6 If resampling methods were used to evaluate model performance, were all model development steps replicated in the resampling process? | Y |
|  |  | 4.7 Was the predictive performance of the model evaluated appropriately—for example, calibration, discrimination, and net benefit? | Y |
| Quality: Concern regarding quality of the analysis | Low Risk | Risk of bias: Risk of bias introduced by the analysis | Low Risk |

Notes: Quality, risk of bias, and applicability were rated as low, high, or unclear risk.

Abbreviations: **Y,** yes; **PY**, probably yes; **PN**, probably no; **N**, no; **NI**, no information; **NA**, not applicable.

**Supplementary Figures**


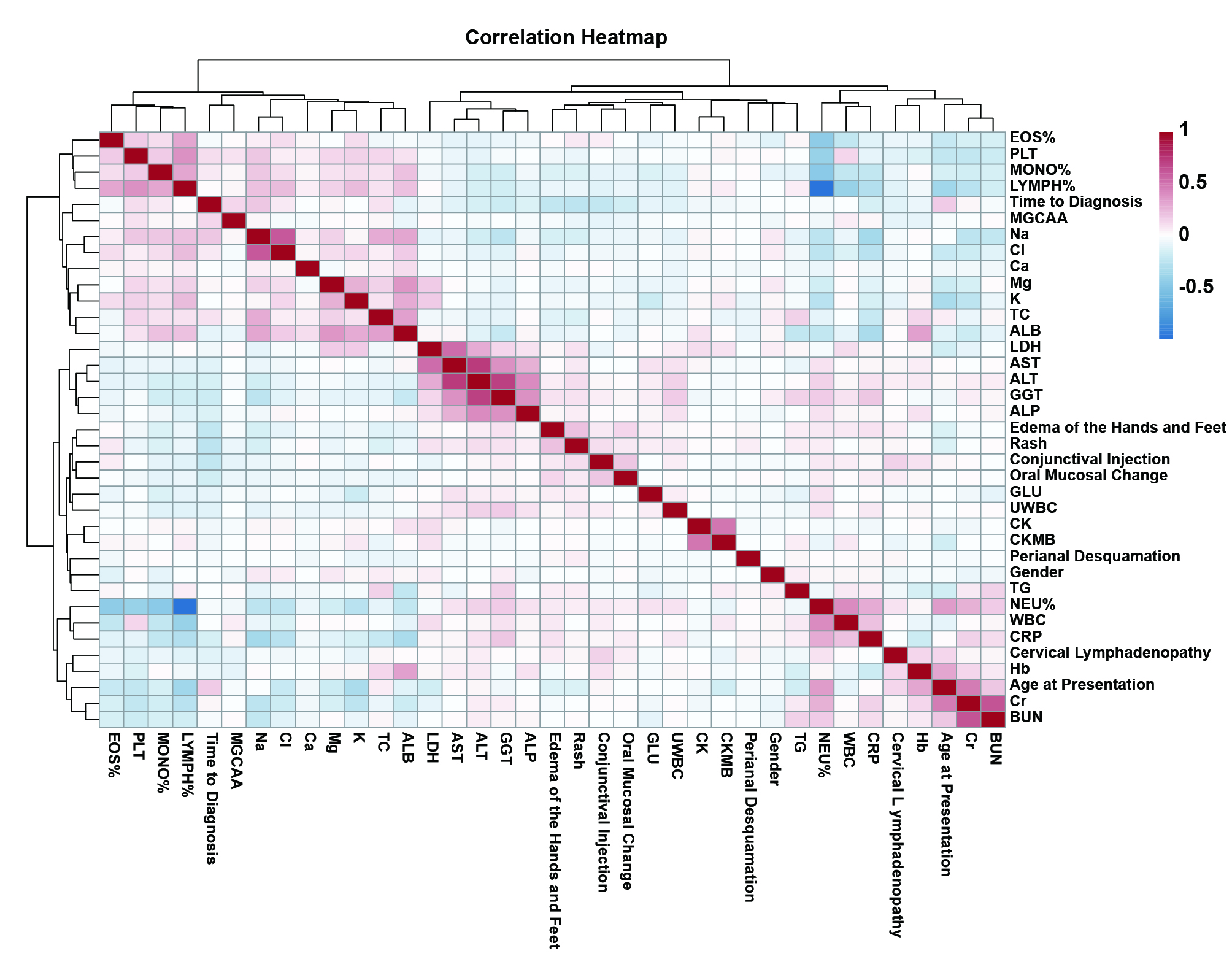


**Supplementary Figure 1.** Correlation heatmap after excluding highly collinear variables and applying multiple imputation.

EOS%: eosinophil percentage; PLT: platelet count; MONO%: monocyte percentage; LYMPH%: lymphocyte percentage; MGCAA: medium-to-giant coronary artery aneurysms; TC: total cholesterol; ALB: serum albumin; LDH: lactate dehydrogenase; AST: aspartate aminotransferase; ALT: alanine aminotransferase; GGT: γ-glutamyl transpeptadase; ALP: alkaline phosphatase; GLU: glucose; UWBC: urinary white blood cell; CK: creatine kinase; CKMB: creatine kinase MB; TG: triglyceride; NEU%: neutrophil percentage; WBC: white blood cell; CRP: C-reactive protein; Hb: hemoglobin; Cr: creatinine; BUN: blood urea nitrogen.


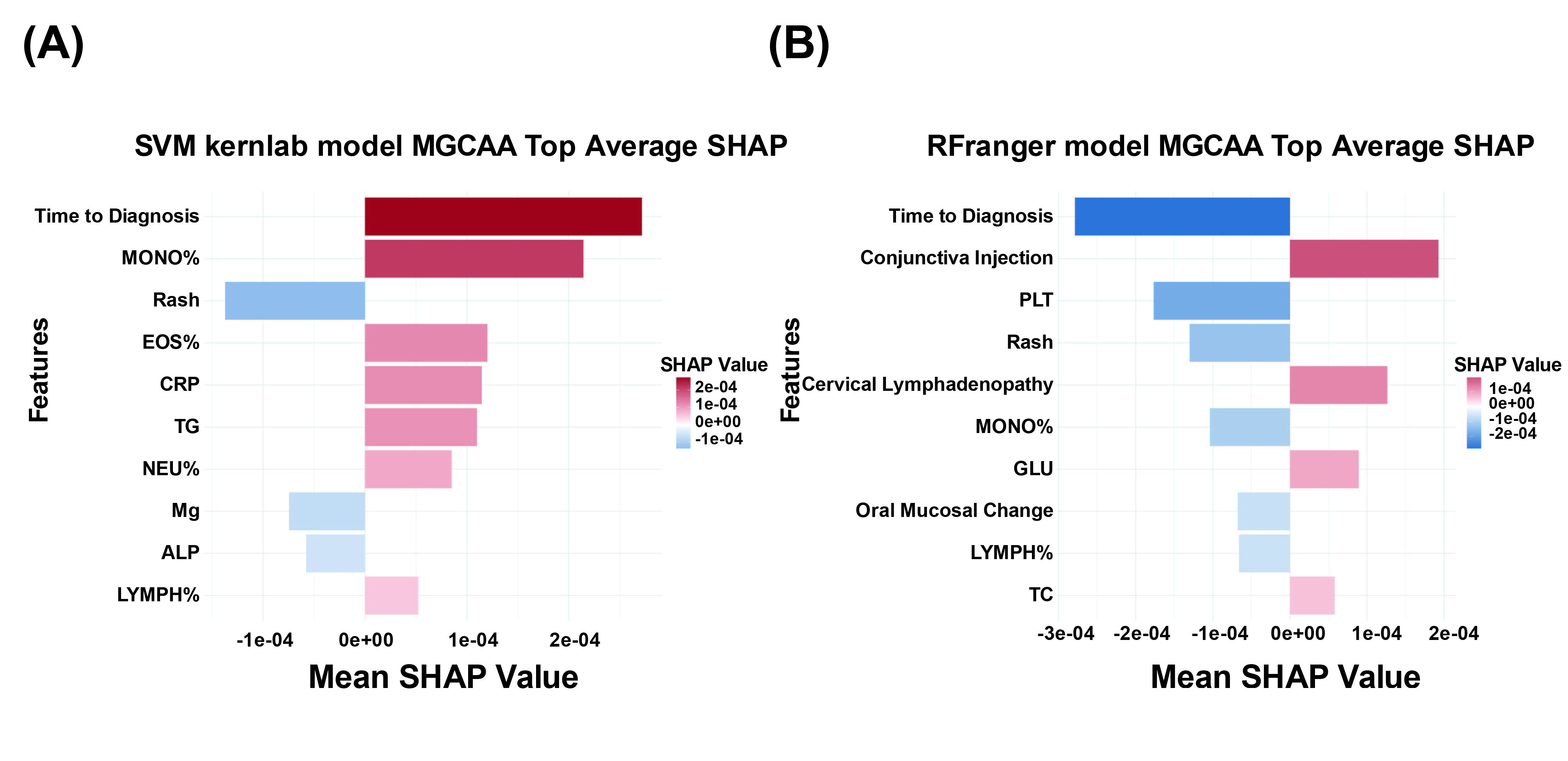


**Supplementary Figure 2.** Top ten features ranked by SHAP values in SVM kernlab and RF ranger models for MGCAA prediction.

(A) The ten most influential features identified in the SVM kernlab model; (B) The ten most influential features identified in the RF ranger model.

MGCAA: medium-to-giant coronary artery aneurysms; MONO%: monocyte percentage; EOS%: eosinophil percentage; CRP: C-reactive protein; TG: triglycerides; NEU%: neutrophil percentage; ALP: alkaline phosphatase; LYMPH%: lymphocyte percentage; PLT: platelet count; GLU: glucose; TC: total cholesterol.


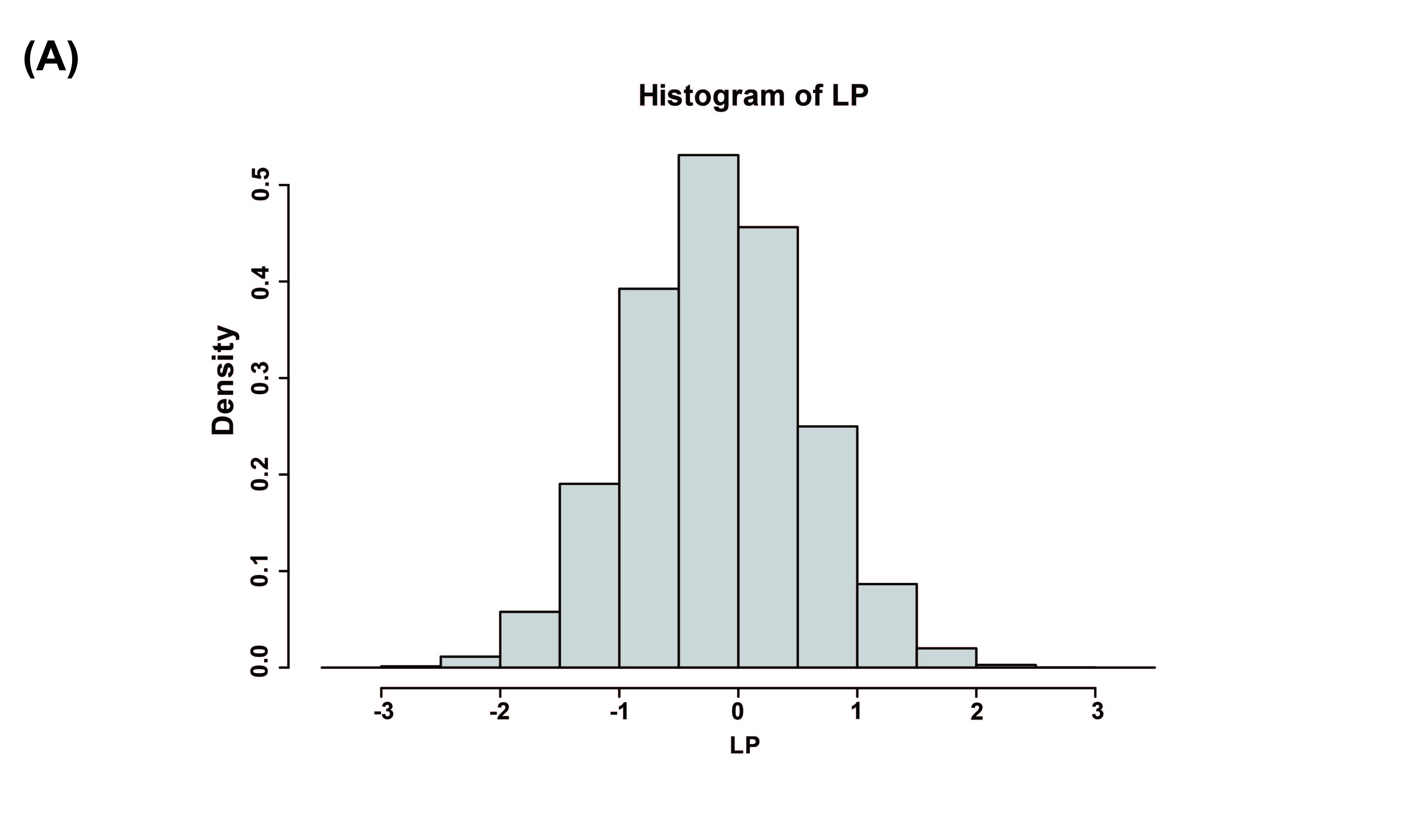


**Supplementary Figure 3**. Histogram of the LP.

LP: linear predictor.

**Reference:**

1. Jone PN, Tremoulet A, Choueiter N, Dominguez SR, Harahsheh AS, Mitani Y, et al. Update on Diagnosis and Management of Kawasaki Disease: A Scientific Statement From the American Heart Association. Circulation (2024) 150:e481-e500. doi:10.1161/cir.0000000000001295

2. McCrindle BW, Rowley AH, Newburger JW, Burns JC, Bolger AF, Gewitz M, et al. Diagnosis, Treatment, and Long-Term Management of Kawasaki Disease: A Scientific Statement for Health Professionals From the American Heart Association. Circulation (2017) 135:e927-e99. doi:10.1161/cir.0000000000000484

3. Dallaire F, Dahdah N. New equations and a critical appraisal of coronary artery Z scores in healthy children. J Am Soc Echocardiogr (2011) 24:60-74. doi:10.1016/j.echo.2010.10.004

4. Austin PC, White IR, Lee DS, van Buuren S. Missing Data in Clinical Research: A Tutorial on Multiple Imputation. Can J Cardiol (2021) 37:1322-31. doi:10.1016/j.cjca.2020.11.010

5. Lunardon N, Menardi G, Torelli N. ROSE: a package for binary imbalanced learning. R J (2014) 6:79-89.

6. Hu J, Xu J, Li M, Jiang Z, Mao J, Feng L, et al. Identification and validation of an explainable prediction model of acute kidney injury with prognostic implications in critically ill children: a prospective multicenter cohort study. EClinicalMedicine (2024) 68:102409. doi:10.1016/j.eclinm.2023.102409

7. Riley RD, Archer L, Snell KIE, Ensor J, Dhiman P, Martin GP, et al. Evaluation of clinical prediction models (part 2): how to undertake an external validation study. BMJ (2024) 384:e074820. doi:10.1136/bmj-2023-074820

8. Moons KGM, Damen JAA, Kaul T, Hooft L, Andaur Navarro C, Dhiman P, et al. PROBAST+AI: an updated quality, risk of bias, and applicability assessment tool for prediction models using regression or artificial intelligence methods. BMJ (2025) 388:e082505. doi:10.1136/bmj-2024-082505
